# Prediction of Pathologic Change Development in the Pancreas Associated with Diabetes Mellitus Assessed by NMR Metabolomics

**DOI:** 10.1021/acs.jproteome.3c00047

**Published:** 2023-04-05

**Authors:** Lenka Michálková, Štěpán Horník, Jan Sýkora, Vladimír Setnička, Bohuš Bunganič

**Affiliations:** † Institute of Chemical Process Fundamentals of the CAS, 165 00 Prague 6, Czech Republic; ‡ Department of Analytical Chemistry, University of Chemistry and Technology, Prague, 166 28 Prague 6, Czech Republic; § Laboratory of NMR Spectroscopy, University of Chemistry and Technology, Prague, 166 28 Prague 6, Czech Republic; ∥ Department of Internal Medicine, 1st Faculty of Medicine of Charles University and Military University Hospital, 169 02 Prague 6, Czech Republic

**Keywords:** pancreatic cancer, NMR metabolomics, diabetes mellitus, prediction of pathologic changes

## Abstract

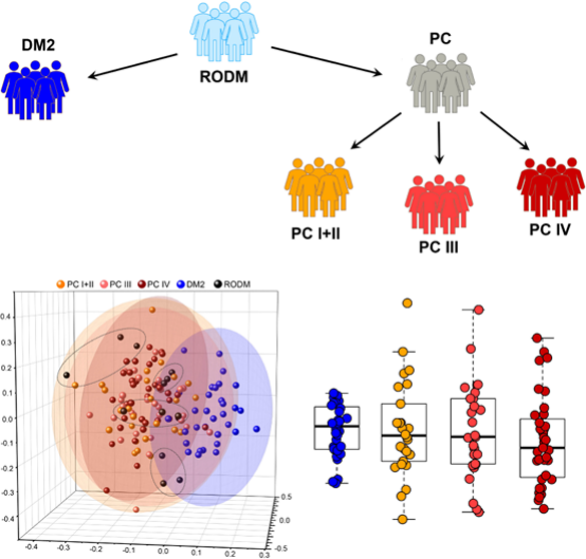

Nuclear magnetic resonance (NMR) metabolomics was used for identification of metabolic changes in pancreatic cancer (PC) blood plasma samples when compared to healthy controls or diabetes mellitus patients. An increased number of PC samples enabled a subdivision of the group according to individual PC stages and the construction of predictive models for finer classification of at-risk individuals recruited from patients with recently diagnosed diabetes mellitus. High-performance values of orthogonal partial least squares (OPLS) discriminant analysis were found for discrimination between individual PC stages and both control groups. The discrimination between early and metastatic stages was achieved with only 71.5% accuracy. A predictive model based on discriminant analyses between individual PC stages and the diabetes mellitus group identified 12 individuals out of 59 as at-risk of development of pathological changes in the pancreas, and four of them were classified as at moderate risk.

## Introduction

Pancreatic cancer (PC) is a very aggressive type of gastrointestinal cancer and is one of the most fatal malignancies. With its increasing incidence and very high mortality, PC ranks as the seventh leading cause of cancer-related death in both genders worldwide, according to the latest global cancer statistics reported in 2020.^1^ The high mortality rate is mostly attributed to late diagnosis due to nonspecific symptoms at early stages. The symptomatology is mainly characterized by loss of appetite, weight loss, digestive problems, impaired glucose tolerance, icterus, abdominal pain, nausea, and fatigue.^2−4^

Current clinical diagnosis relies on high-resolution imaging methods, such as computed tomography (CT), magnetic resonance imaging (MRI), endoscopic retrograde cholangiopancreatography (ERCP),^5^ and endoscopic ultrasound (EUS) with tissue biopsy. The use of commonly known markers of PC, serum carbohydrate antigen 19-9 (CA 19-9) and carcinoembryonic antigen (CEA), has a number of limitations, most notably insufficient sensitivity and specificity.^6^ A positive diagnosis usually comes at later stages of PC.

According to the Classification of Malignant Tumors (TNM) provided by the Union for International Cancer Control (UICC),^7^ the cancer severity is assessed by the tumor size (T), number of attacked lymph nodes (N), and presence of metastasis (M). In this manner, four fundamental stages are defined (I–IV), which can be further subdivided (Table 1). Unfortunately, due to late diagnosis, only one in five diagnosed PC cases is a nonmetastatic stage with local tumor, enabling surgery followed by adjuvant chemotherapy.^8,9^

**Table 1:** Classification of Malignant Tumors Tumor Size (T), Number of Attacked Lymph Nodes (N), and Presence of Metastasis (M)

stage	T	N	M
Ia	≤2 cm (T1)	none (N0)	none (M0)
Ib	2–4 cm (T2)	none (N0)	none (M0)
IIa	>4 cm (T3)	none (N0)	none (M0)
IIb	T1–T3	1–3 regional lymph nodes (N1)	none (M0)
III	T1–T3	>4 regional lymph nodes (N2)	none (M0)
	tumor of any size affects truncus coeliacus, a. mesenterica superior, a. hepatica communis (T4)	any N	none (M0)
IV	any T	any N	metastasis (M1)

New sensitive diagnostic tools are therefore highly demanded for early PC diagnosis and differentiation of its clinical stages. One of the modern approaches used recently is metabolomics, which can be defined as a comprehensive study of the metabolites present in any biological sample. Bodily fluids are an example of such samples. In general, bodily fluids are easily accessible, their sampling is noninvasive and cost-effective, and, on top of that, they are enriched with metabolites that can serve as a potential source of cancer diagnostic and predictive and prognostic biomarkers. Metabolomics is a powerful method for the identification of such biomarkers for interpreting biological pathways affected by the disease and assessment of function of complex biological systems.^2,10^ Typically employed analytical techniques include nuclear magnetic resonance (NMR) spectroscopy and mass spectrometry (MS). Regarding PC, a number of metabolomic studies^11−19^ have already shown differences in metabolite levels between PC patients, healthy controls, and patients with other conditions like chronic pancreatitis, diabetes mellitus, or other cancers. The combination of novel circulating biomarkers determined at omic levels with clinical data might be the future strategy for the early diagnosis of PC.^2^ Besides early diagnosis and monitoring of PC progression, differentiation of individual clinical stages could facilitate decision making for surgical treatment. Such studies have been done so far solely on mouse models showing, e.g., different metabolic patterns in blood serum of early- or late-stage pancreatic ductal adenocarcinoma^20^ or PC development across different tumor phenotypes.^21^

In our previous work, NMR metabolomics was employed for prediction of future development of PC in recently diagnosed diabetes mellitus patients using blood plasma samples.^12^ Here, the number of PC samples was significantly increased, allowing a subdivision of the group according to individual PC stages. The discriminant analyses between individual PC stages and samples of diabetes mellitus patients were then used for construction of predictive models for finer classification of at-risk individuals.

## Materials and Methods

### Subject Recruitment and Plasma Sample Collection

This study followed the Helsinki Declaration and the Collection of Laws of the Czech Republic and was approved by the Ethics Committee at the Military University Hospital Prague (108/8-44/2015). All enrolled subjects of this study signed informed consent.

Participants of this study were recruited at the Department of Gastrointestinal Endoscopy at the Internal Clinic of the Military University Hospital and the First Faculty of Medicine of Charles University in Prague. This study expanded on our previous article;^12^ plasma samples were collected from healthy control (HC) subjects, pancreatic cancer (PC) patients, type 2 diabetes mellitus (DM2) patients, and recently diagnosed diabetes mellitus (RODM) patients.

PC patients were divided into three groups according to clinical stages following the TNM classification: stage IV, stage III, and early stages, combining stages Ib, IIa, and IIb to ensure a balanced group size for statistical analysis. The stage of PC patients was determined by standard examination by EUS, MRI, and/or CT and by histopathological analysis. The study design attempted to balance the biological characteristics of the enrolled participants, as shown in Table 2. All groups included subjects in the same age range as confirmed by the *F*-test. However, *t*-test analyzing the equality of means did not confirm the equality in the case of healthy controls when compared to other groups. Similarly, a group of DM2 patients revealed significantly raised values of BMI. A gender imbalance was identified only between PC III and HC groups by the X^2^-test of independence. A statistical significance of age, sex, and BMI in pair discriminations can be seen in Table S1. Nevertheless, due to a limited number of samples, any possible influence of different biological characteristics among groups had to be omitted. All samples received from the hospital were used in the study providing 207 samples altogether.

**Table 2:** Overview of the Groups Studied

			age, years		sex		BMI
group		no. of samples	range	mean	female	male	mean ± SD
HC		28	49–85	62	18	10	24.9 ± 3.0
DM2		32	50–82	68	16	16	30.3 ± 6.3
PC	Ib	4	49–75	66	2	2	27.0 ± 4.4
	IIa	9	49–85	68	4	5	27.5 ± 5.5
	IIb	13	51–78	71	8	5	25.7 ± 4.8
	I + II	26	49–85	69	14	12	26.5 ± 4.9
	III	27	50–82	69	8	19	25.6 ± 4.1
	IV	35	49–83	67	17	18	24.4 ± 3.1
RODM		59	50–85	65	32	27	28.4 ± 4.6

Sterile blood collection tubes with K_3_EDTA as an anticoagulant were used for collection of venous blood (9 mL). Besides NMR metabolomic analysis, blood samples were also subjected to a routine biochemical test, assessing basic physiological indicators, such as fasting plasma glucose (FPG), glycated hemoglobin (Hb_1c_), human serum albumin (HSA), and protein or cancer biomarker (CA 19-9, CEA) levels. The biochemical characteristics are summarized in the Supporting Information (Table S2). The plasma fractions for NMR analysis were obtained by centrifugation at 15 000*g* for 10 min and frozen immediately afterward (−80 °C).

### Sample Preparation

The plasma samples were thawed at room temperature. Low-molecular-weight plasma fraction was obtained by centrifugation through an Amicon 3-kDa cutoff filter (Merck, Germany) of 300 μL plasma for 30 min at 12 000 rpm. The following step was mixing the ultrafiltrate with 300 μL of phosphate buffer in D_2_O (0.1 mol·L^–1^; pH = 7.4; 0.1 mmol·L^–1^ sodium salt of trimethylsilyl-[2,2,3,3-*d*_4_]-propionic acid (TSP); 38 mmol·L^–1^ NaN_3_). The prepared mixture (600 μL) was transferred into a 5 mm NMR tube for the analysis.

### Data Acquisition

One-dimensional proton NMR spectra were acquired using a Varian INOVA 500 MHz spectrometer (Varian Instruments Inc.) operating at 499.87 MHz proton Larmor frequency equipped with the probe with an inner ^1^H coil for higher sensitivity. Prior to measurements, samples were kept for at least 10 min inside the NMR probe for temperature equilibration (298.15 K). For each plasma sample, a water-suppressed Carr–Purcell–Meiboom–Gill (CPMG) spin echo pulse sequence with 1000 scans, 65 536 data points over a spectral width of 8000 Hz, with a relaxation delay of 2 s, a saturation delay of 2 s, and an acquisition time of 2.7 s was used to acquire the ^1^H NMR spectra.

### Data Processing

The Fourier transform spectra were manually corrected for phase and baseline distortions using Chenomx NMR Suite 8.0 (ref (22), NMR Suite program, Canada). The experimental spectrum was referenced to TSP. The solvent/water signal residuum was subtracted. Finally, TSP signal linewidth was determined, and pH was set to 7.4 according to the used buffer.

Untargeted profiling was performed in the Chenomx Profiler. In all samples, 63 metabolites were quantified according to precise fitting and known concentration of the chemical shift standard TSP. Two metabolites were removed from the metabolic profile subjected to statistical analysis: glycerol, as it is present in the membrane of the filters used, and propylene glycol, as an environmental contaminant. The obtained metabolic profiles are represented by a concentration of 61 assigned compounds normalized to total area to suppress differences caused by plasma dilution. A complete list of metabolites assigned, including ^1^H NMR signals used for quantification, can be found in the Supporting Information (Table S3).

### Statistical Analysis

**Figure 1:**
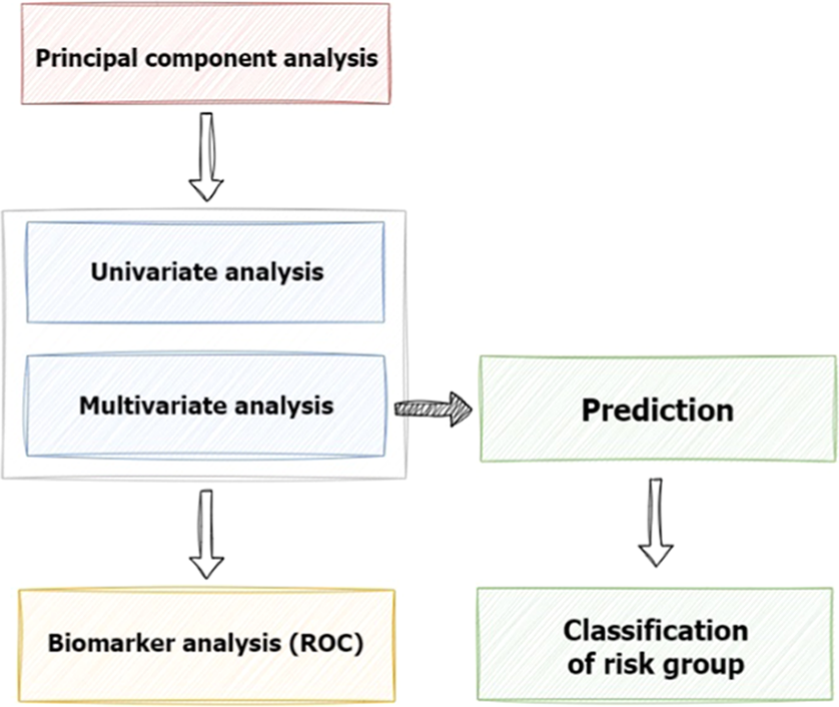
Schematic depiction of the statistical analysis used within this work.

A flow of statistical analysis applied to data obtained within this work can be seen in Figure 1.

A principal component analysis (PCA) as an unsupervised method is used for initial exploratory analysis to inspect the definition of each group, its homogeneity and check for outlying samples. Subsequently, orthogonal partial least-squares discriminant analysis (OPLS-DA) was applied for discrimination of each two groups (HC, DM2, and PCs). Each model retained the minimum number of components with maximized accuracy. For indicative discrimination of 3 groups, PLS-DA was used. For validation of each model, 100 cycles of a Monte Carlo cross-validation scheme were applied. In detail, 90% of the samples was randomly selected at each iteration as a training set to build the model; the remaining 10% were subsequently tested on performance characteristics for the classification. Furthermore, the permutation test was performed on randomly generated groups for each OPLS-DA model. In the permutation test, identical data set was used with randomly reassigned group labels. The process of relabeling was repeated 1000 times to estimate the distribution of the performance measures. The obtained *P*-value was calculated as a number of randomly labeled groups with better model characteristics than the original data divided by a number of trials, in this case 1000. The permutation test was conducted for all performed OPLS-DA models. Subsequently, a univariate analysis was performed. A statistical significance was assessed by the nonparametric Wilcoxon rank-sum test following the biological assumption that metabolites are not normally distributed. The obtained *P*-values were adjusted using the Benjamini and Hochberg correction (ref (23)). According to the general metabolomics rule, the threshold of adjusted *P*-values was set to <0.05 for statistical significance. Additionally, fold change analysis was performed to observe the trend of the metabolite variations within each discrimination. All above-mentioned statistical analyses were performed on a complete list of metabolites determined using the open-source software R (ref (24)).

According to metabolites found with statistical significance for each discrimination, a potential panel of biomarkers was proposed. Metaboanalyst 4.0 (ref (25)) was used for the performance assessment of a potential biomarker panel by construction of a receiver-operating characteristic (ROC). Selected algorithm for ROC analysis was random forest. Results were averaged according to 100 cross-validations. The biomarker panel performance was evaluated by comparison of area under the receiver-operating curve (AUC) accompanied with a 95% confidence interval (CI). Generally, ROC analysis is considered the standard method for medical diagnostic tests, and the utility of a biomarker with an AUC above 0.8 is acceptable.^26^ In total, 3 biomarker panels were tested for discrimination between individual PC stages and both control groups: panel containing all 61 metabolites (i), panel consisting of 9 metabolites with statistical significance found across all above-mentioned discriminant analyses (ii), and panel of 8 metabolites identified previously in discrimination between generally defined PC group and both controls (iii).

Finally, the OPLS discriminant analyses of individual PC stages and the DM2 group were used for classification of RODM samples. For construction of such predictive models, all metabolites were used. Overall, 60 prediction models were created for RODM classification, and 20 models for each PC stage/DM2 discrimination. The models utilized all samples from both groups; however, different samples were taken for training and validation set using a Monte Carlo cross-validation scheme. For RODM classification, only models with accuracy above 80% were utilized. To ensure a balanced group size for classification, a random selection of 25 from 59 RODM samples was provided by function *sample* in R (package *base*).^24^ The final classification considers the ratio between the number of PC classifications of a given sample and the number of its applications in the prediction model.

Visualization of the results was performed using the open-source software R (ref (24)), Metaboanalyst 4.0 (ref (25)), and Origin (ref (27)), and superposition of 3D score plots was performed in DS visualizer (ref (28)).

## Results and Discussion

In this study, more than 200 blood plasma samples were evaluated. Similarly to the previous paper,^12^ the main objective remains to compare metabolic profiles of pancreatic cancer (PC) patients to those of diabetes mellitus patients (DM2) and healthy control (HC). The observed differences can then be used to construct a prediction model for classification of recent-onset diabetes mellitus (RODM) patients and their future development.

The number of analyzed samples remained similar in each group as in the previous publication except for the group of pancreatic cancer patients, which was more than doubled. The increase in the number of samples in the PC group enabled its subdivision into three groups, according to the severity of the diagnosis. The PC early stages were clustered together, providing 26 samples of stages I and II (group PC I + II). The stage III group consisted of 27 samples (PC III) and stage IV of 35 samples (PC IV). The definition of individual groups and detailed information can be found in the Materials and Methods section. Altogether, 88 plasma samples of pancreatic cancer patients were analyzed by ^1^H NMR spectroscopy. Subsequently, the untargeted profiling provided a concentration profile of 61 low-molecular metabolites that served as an input into statistical analysis.

Initially, the unsupervised statistical analysis (PCA) was performed on each group to examine the definition and homogeneity of the group. No outliers were observed and/or excluded. Subsequently, the metabolic profiles of all three PC groups were compared to those of healthy controls (HCs, 28 samples) and of diabetes mellitus patients (DM2, 32 samples) via OPLS discrimination analysis. The analysis showed that samples from all three clinical stages of pancreatic cancer can be differentiated from those of both HC and DM2 groups with satisfactory performance values. The results are summarized in Table 3, and the OPLS-DA score plots can be found in Figure S1.

**Table 3:** Performance Characteristics of OPLS-DA between PC Stage Groups and Both Control Groups^a^

discrimination	components no.	accuracy, %	sensitivity, %	specificity, %	*P*-value
HC vs PC I + II	5	85.6	83.4	87.8	<0.001
HC vs PC III	4	83.5	76.7	90.1	<0.001
HC vs PC IV	5	89.5	88.4	90.9	<0.001
DM2 vs PC I + II	8	82.3	77.5	86.3	<0.001
DM2 vs PC III	8	83.0	79.7	85.8	<0.001
DM2 vs PC IV	8	84.6	82.9	86.5	<0.001

a *P*-values were obtained by a permutation test.

Subsequent univariate analysis revealed statistically significant metabolites (*P*-value <0.05) for discrimination of PC stages from HC and DM2 groups, respectively (see Table S4). Trends of metabolic changes were also followed by fold-change analysis (see Table S5). Among the metabolites significant for each discrimination analysis, nine metabolites met the statistical significance in all cases (Table 4), namely, 3-hydroxyisovalerate, creatine, fumarate, gluconate, lysine, mannose, N-acetylcysteine, proline, and propionate. When the discrimination is repeated just using this set of metabolites, the performance values are comparable or even better to the complete profile of 61 metabolites as assessed by receiver-operating characteristic (ROC) curves generated by the random forest algorithm (see Table S6). The found statistical significance of four metabolites (creatine, lysine, mannose, and proline) is consistent with the original biomarker panel proposed in the previous publication. Originally, eight metabolites were selected for simultaneous discrimination of generally defined PC and both control groups.^12^ The other four metabolites (3-hydroxybutyrate, alanine, glutamate, and valine) were found more significant for late stages of pancreatic cancer (Table 4, last four rows). Although their concentration levels found in all PC stages differ substantially from those found in samples of both HC and DM2, their significance rises with the PC stage (see Figure S2). It is worth noting that the original biomarker panel also provides satisfactory AUC values (see Table S6).

**Table 4:** Overlap of Significant Metabolites According to Wilcoxon Rank-Sum Test, *P*-Value < 0.05^a^

	PC vs HC			PC vs DM2		
metabolite	I + II	III	IV	I + II	III	IV
3-hydroxyisovalerate	0.0211	0.0377	0.0190	0.0231	0.0452	0.0187
creatine^b^	0.0001*	0.0001*	0.0001*	0.0003*	0.0006*	0.0004*
fumarate	0.0026	0.0014	0.0005*	0.0122	0.0160	0.0052
gluconate	0.0285	0.0039	0.0003*	0.0003*	0.0000*	0.0000*
lysine^b^	0.0000*	0.0000*	0.0004*	0.0059	0.0055	0.0269
mannose^b^	0.0000*	0.0001*	0.0000*	0.0004*	0.0041	0.0000*
N-acetylcysteine	0.0047	0.0007*	0.0009*	0.0428	0.0123	0.0204
proline^b^	0.0187	0.0100	0.0313	0.0059	0.0032	0.0187
propionate	0.0147	0.0279	0.0089	0.0004*	0.0032	0.0000*
3-hydroxybutyrate^b^	0.0085	0.0008	0.0001*	0.0030	0.0304	0.0950
alanine^b^	0.1401	0.1352	0.0002	0.1651	0.2362	0.0021
glutamate^b^	0.0497	0.2666	0.0792	0.1598	0.3952	0.1838
valine^b^	0.4274	0.3973	0.0444	0.7576	0.8943	0.2436

a * refers to *P*-value < 0.001.

b Original biomarker panel associated with generally defined pancreatic cancer (ref (12)).

**Figure 2:**
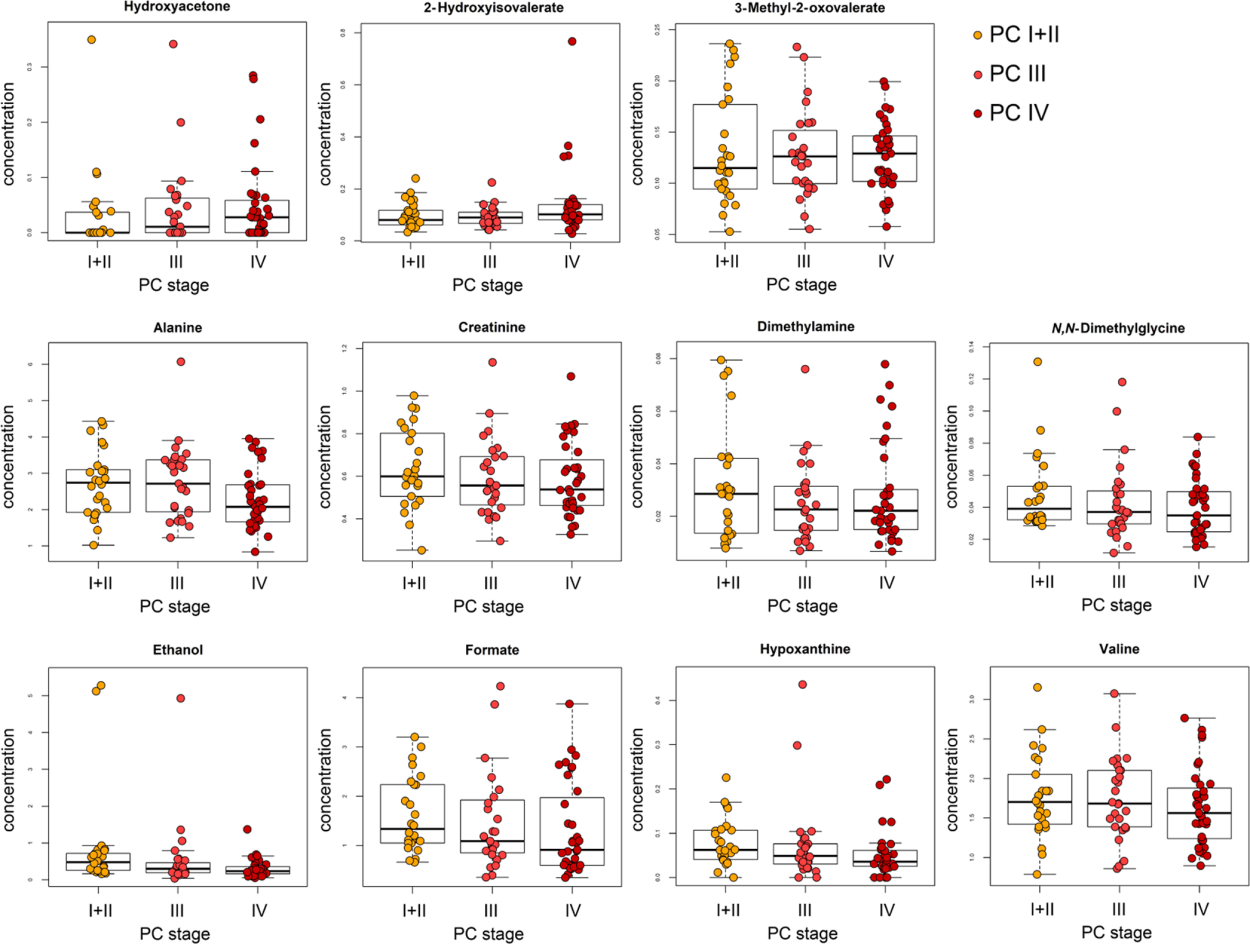
Box plots of metabolites gradually changing the concentration levels with the PC stage. No statistical significance was found in discrimination between any pair of PC stages.

The metabolites, statistically significant for simultaneous discrimination of PC stages from HC and DM2 groups, can be characterized by similar concentration levels across all three PC stages, which differ noticeably from those found in the HC and DM2 groups. When focusing on differences in concentration levels among individual PC stages, there are a certain number of metabolites gradually changing their concentration with the PC stage, reflecting the progress of the disease. The concentration of hydroxyacetone, 2-hydroxyisovalerate, and 3-methyl-2-oxovalerate gradually increases with the PC stage, while the concentration of alanine, creatinine, dimethylamine, *N*,*N*-dimethylglycine, ethanol, formate, hypoxanthine, and valine decreases (Figure 2). However, a univariate statistical analysis, a Wilcoxon rank-sum test, did not reveal any statistically significant metabolites in discrimination between any pair of PC stages.

The found changes in metabolite levels can be attributed predominately to the well-known biological processes associated with cancer cell growth and proliferation.^29,30^ Namely, the increased energy demand is being satisfied by elevated glycolytic metabolism in the hypoxic tumor environment, the Warburg effect.^31,32^ Glycolytic intermediates then enter and affect multiple pathways generating nucleotides, lipids, and amino acids. Furthermore, cancer is manifested by increased blood levels of ketone bodies and decreased concentrations of amino acids, both also involved in altered energy metabolism. Similarly, the metabolism of branched-chain amino acids is disrupted, as observed from increased levels of energy balance markers.^29,30,33,34^

**Figure 3:**
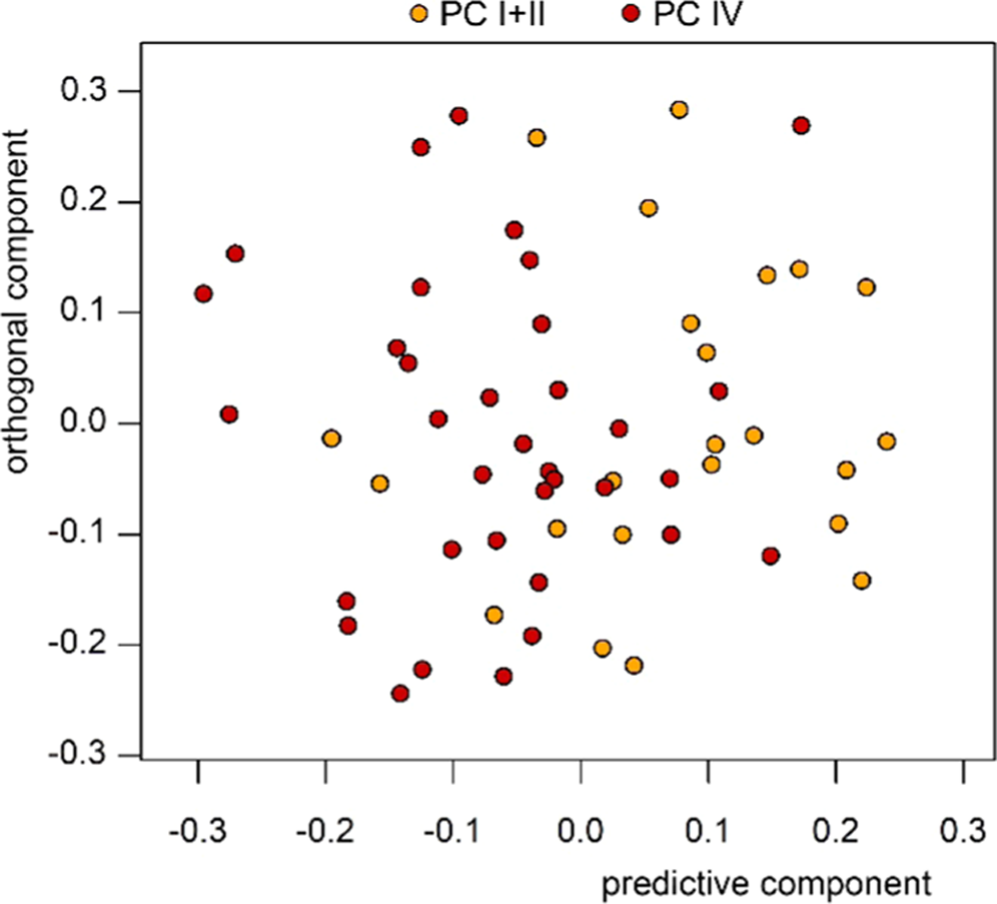
OPLS-DA of PC I + II (*n* = 26, orange circles) and PC IV (*n* = 35, red circles) using 61 normalized metabolites; Acc. 71.5%, Sen. 73.9%, and Spe. 68.3%.

Similarly, as with univariate statistical analysis, neither unsupervised PCA nor supervised PLS discriminant analysis of the three groups corresponding to the PC clinical stages revealed any clustering trend (see Figures S4 and S5). Subsequent OPLS discriminant analysis was only partially successful, as the metabolic changes among clinical stages are very subtle, as mentioned above. The discriminant models were found satisfactory only for discrimination of early-stage PC I + II from late-stage PC IV. The accuracy reached 71.5% using the six-component model (Figure 3), and the *P*-value obtained by a permutation test reached 0.149. The middle-stage PC III cannot be discriminated either from early- or late-stage PC reaching *P*-values 0.994 and 0.215, respectively.

The metabolic changes describing the PC development are much less pronounced than the comparison to the control group. Similar results have also been obtained by other groups examining metabolic differences of PC progression in humans. Obviously, the discrimination of individual cancer stages is much more challenging. Tumas et al.^35^ analyzed changes in concentration levels of amino acids in PC plasma samples via univariate statistics. The diminishing levels from stage I toward stage IV were observed only for few amino acids, while the others possessed a U-shape curve across PC stages. A U-shape concentration profile across stages has an adverse effect on discrimination analysis when different stages are included within one group. Obviously, a similar effect can be introduced to the statistical analysis by clinical misclassification of the PC stage of a patient. Unfortunately, finer splitting of the early PC stages in our case can be misleading due to the imbalanced number of samples in each group. The boxplots of concentration levels with fine splitting of PC stages can be found in Figure S3. Other metabolomic studies were focused mainly on differentiation of metastasis stage. Results of a recent LC-TQ/MS study of blood plasma also demonstrated difficulties in identification of metabolic changes to distinguish PC cases in progressive stage I, stage II, stage III, and stage IV. Only subtle changes were observed, and only succinate and gluconate levels were correlated to PC metastasis.^31^ Multiple metabolite changes between PC patients and healthy controls were also observed in the GC/MS metabolomic study of serum samples.^15^ However, subsequent differentiation between late stages of PC was performed in a rather tentative manner due to the limited number of samples used in the study.

Other studies combine the found metabolic profile with levels of serum carbohydrate antigen (CA 19-9).^36−38^ On the one hand, CA 19-9 is the most common tumor marker assessed in pancreatic cancer;^39^ on the other hand, CA 19-9 values can be excessively increased due to cholestasis, pancreatitis, and other chronic reasons.^40,41^ In this study, a correlation between CA 19-9, PC stage, and metabolite levels was also attempted. Two cutoff limits of CA 19-9 were used (100 and 700 kU/L), providing two data sets: one operating only with 100 kU/L, which divided data into two groups, and the other utilizing both cutoff limits, which provided three groups. Initially, CA 19-9 levels were projected into original color-coded PCA analysis (see Figure S6). Unfortunately, no obvious clustering trend was observed. Similarly, the discrimination analysis of pancreatic cancer stages did not improve by employing CA 19-9 as an input parameter. Therefore, our findings tend to agree with Moore’s study,^41^ where no straight correlation of CA 19-9 with PC progression was observed, and CA 19-9 levels should be interpreted with caution, mainly in the clinical context of individual cases.

Although individual PC stages could not be discriminated from each other with reasonable probability levels, the alterations of plasma metabolite can still be utilized in clinical diagnosis. We showed recently that discrimination of PC and DM2 groups can be taken as a prediction model for the identification of persons at high risk of future PC development from the cohort of recently diagnosed diabetes mellitus patients. The relationship between pancreatic cancer and diabetes mellitus or impaired glucose tolerance had been observed in 80% of PC patients.^42^ The specific type of diabetes associated with PC is called pancreatogenic diabetes (T3cDM), and it is often misdiagnosed as type 2, the most prevalent type, due to similar development.^43−45^ The identified at-risk patients should then undergo a detailed screening and medical surveillance.

In our previous work, six RODM patients were identified as at-risk of future development of PC. A subsequent physical examination revealed significant changes in pancreas pathology in four of them. The prediction model was based on discrimination of DM2 and a generally defined PC group, into which RODM patients were classified. Here, three groups of PC according to stage are at our disposal for construction of a finer prediction model. Similarly, as with the procedure described in our previous work, data of 59 RODM patients were repeatedly assigned to OPLS-DA discrimination of individual PC stages and the DM2 group.

Complete metabolic profiles of 61 metabolites normalized to total sum were used for construction of prediction models. Twenty models were created for discrimination of each PC stage and DM2, resulting in a total of 60 prediction models (for details, see Materials and Methods). A random selection of 25 RODM samples for each classification was used to ensure a balanced group size. Only models with an overall accuracy of above 80% were used for RODM classification. The final classification is expressed as a percentage of the sample classification into a given PC stage vs the number of sample applications in the models (Table 5).

**Table 5:** RODM Sample Classification as PC According to Prediction Models Based on OPLS-DA between PC Stages and DM2 and between PC I + II and PC IV Expressed as a Ratio of Sample Classification as PC vs Sample Applications in Predictive Models

	prediction model						
sample	DM2 vs PC I + II		DM2 vs PC III		DM2 vs PC IV		PC I + II vs PC IV
RODM 001	0%	0/12	0%	0/10	100%	9/9	IV
RODM 008^a^	0%	0/8	100%	11/11	71.4%	5/7	IV
RODM 011^a^	0%	0/7	0%	0/13	100%	11/11	IV
RODM 015^a^	0%	0/7	0%	0/6	100%	11/11	IV
RODM 032	0%	0/7	0%	0/10	100%	8/8	I + II
RODM 036^a^	0%	0/11	0%	0/7	100%	11/11	IV
RODM 037	0%	0/9	0%	0/10	100%	7/7	IV
RODM 048	92.3%	12/13	100%	12/12	100%	9/9	I + II
RODM 061	100%	8/8	100%	10/10	38.5%	5/13	I + II
RODM 065	83.3%	5/6	66.7%	6/9	100%	8/8	IV
RODM 066	0%	0/8	0%	0/8	81.8%	9/11	I + II
RODM 071^a^	88.9%	8/9	100%	7/7	93.7%	15/16	I + II

a Patients identified as at-risk of future development of PC in the previous work (ref (12)).

Four out of 59 total RODM samples were classified as PC using a PC I + II/DM2 discrimination model, namely, RODM 048, RODM 061, RODM 065, and RODM 071. Surprisingly, the same four samples were classified as PC also using a PC III/DM2 discrimination model. Additionally, sample RODM 008 was selected; altogether, five samples were classified as PC. The prediction model based on PC IV/DM2 discrimination selected 12 RODM samples; however, five of them were already classified as PC in the preceding classifications. The whole list of samples classified into individual PC stages can be seen in Table 5. The current selection of at-risk RODM patients is highly consistent with the original classification done on general PC and DM2 groups. Five out of six at-risk RODM patients were selected also using current models, reflecting fine PC group splitting. Only RODM 003 sample was not classified with the current models. It is worth noting here that no changes in pancreas pathology were found in this patient during the last health condition re-examination.

**Figure 4:**
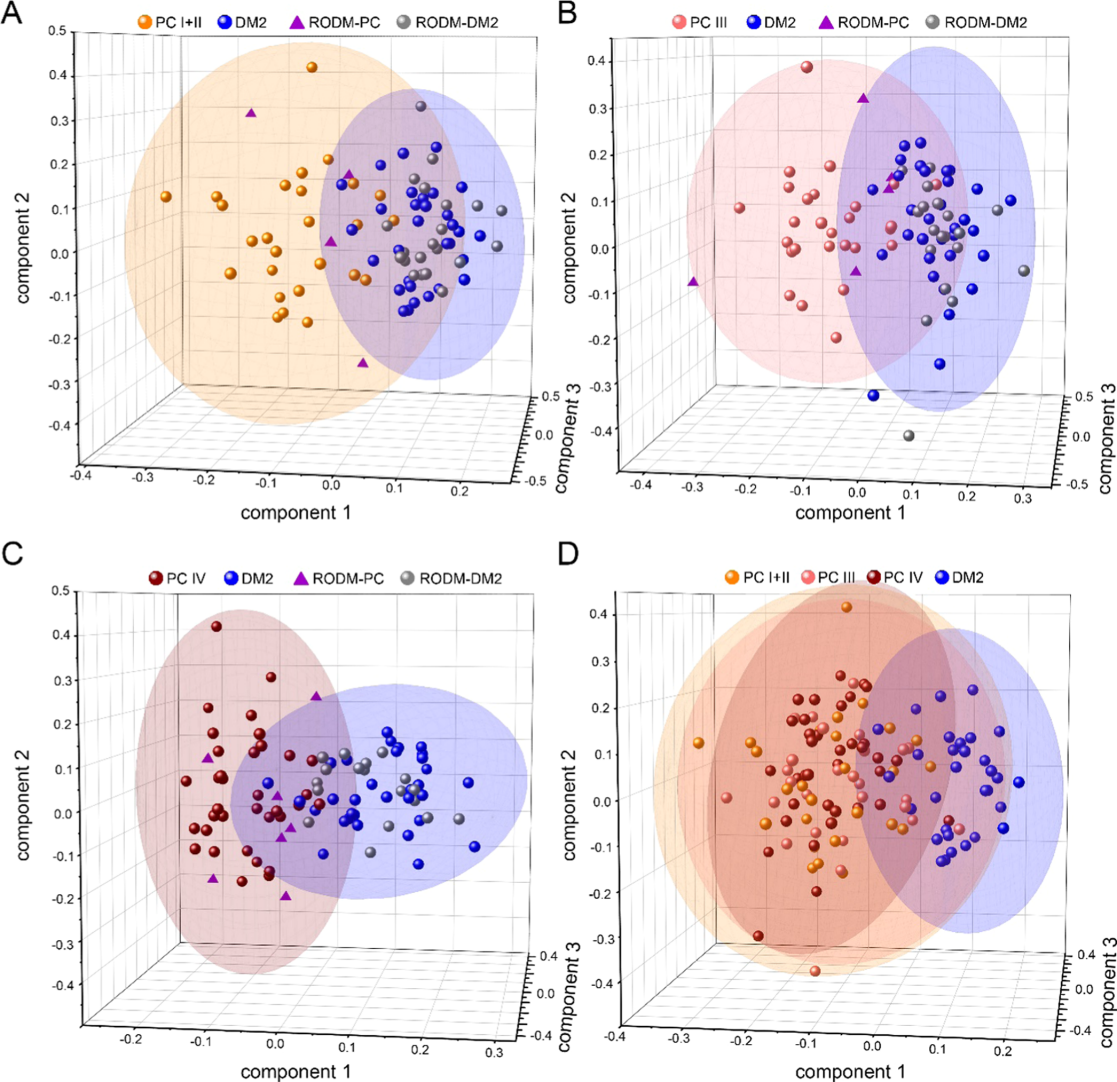
Score plot of the first 3 components of the OPLS-DA prediction model based on PC I + II and DM2 discrimination (A), PC III and DM2 discrimination (B), and PC IV and DM2 discrimination (C). A superposition of the three discriminant analyses (D).

An example of a single RODM group classification into PC stages can be seen in 3D score plots defined by the first three components of the OPLS discriminant analysis (Figure 4A–C). The RODM group consisted of 25 samples; among them, four RODM samples assigned as PC in all three stages, sample RODM 008 classified as PC in PC III and PC IV stages, and also three RODM samples selected only by the PC IV model. Interestingly, OPLS-DA places PC groups to the same space relative to the DM2 group position. Figure 4D shows the three discriminant models used put into a single 3D plot. The three OPLS models were overlaid using positions of the four most outlying DM2 samples. In particular, Figure 4D shows PC III and PC IV samples positioned in PC I + II/DM2 discriminant plot and relevant 95% confidence intervals (CI) of all groups concerned. The CI of PC IV and DM2 occupies a smaller space than the other PC groups, indicating their better definition and resulting homogeneity. The fact that all three PC groups occupy a rather similar space illustrates in a different way the difficulty in discrimination between individual PC stages using the current data set.

**Figure 5:**
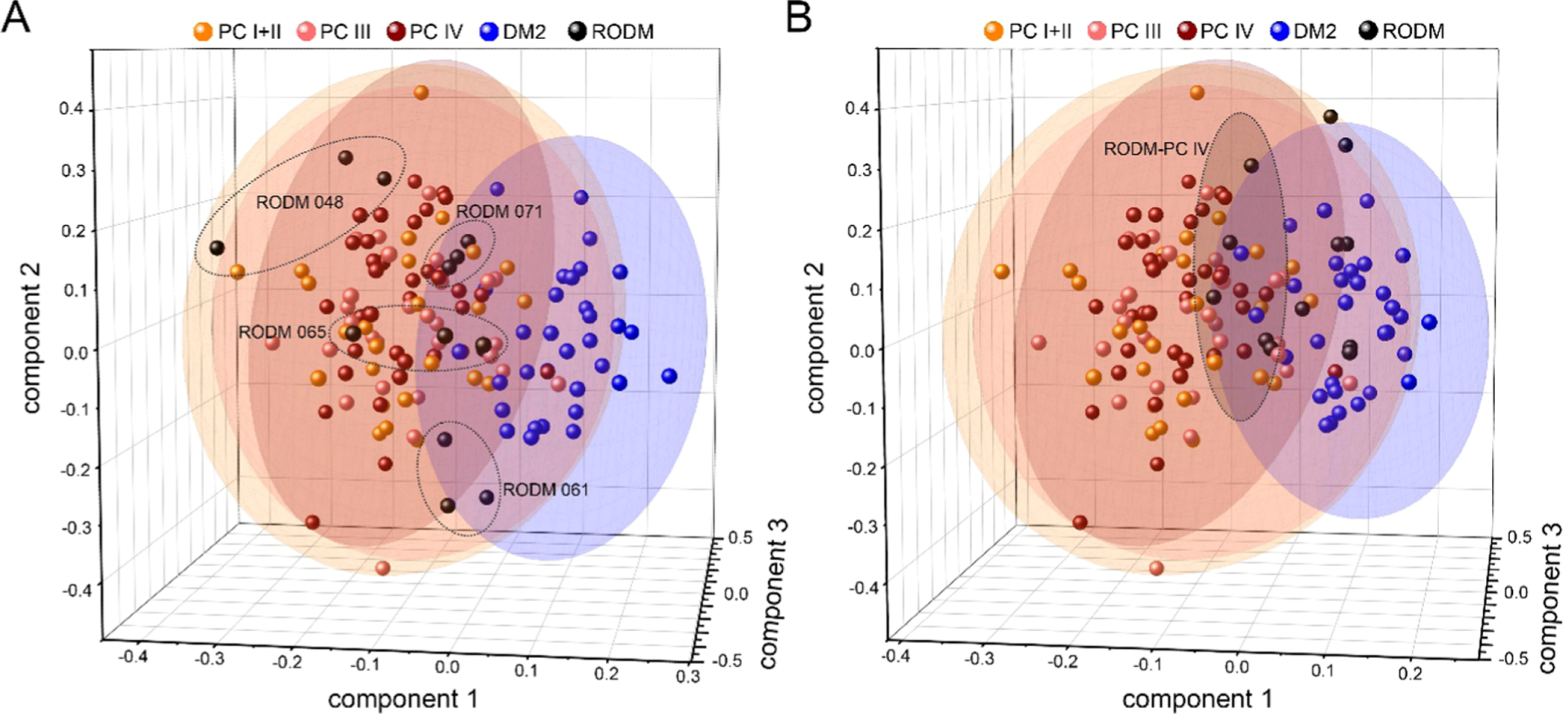
Superposition of the three discriminant analyses showing the classification of four RODM samples classified as PC in all cases (A) and the classification of four RODM samples classified as PC IV (B).

The superposition of the three discrimination models enables mutual comparison of individual RODM sample classification and the behavior of each sample in different models. Figure 5A shows the positioning of the four RODM samples that are classified as PC in all cases. Their location in the 3D space does not differ significantly from one discriminant analysis to another, hitting predominately the intersection of all three 95% CI. On the contrary, the RODM samples assigned as PC only in the discrimination of later stage are spread across the DM2 group and hit the PC space only in PC IV discrimination (Figure 5B).

**Figure 6:**
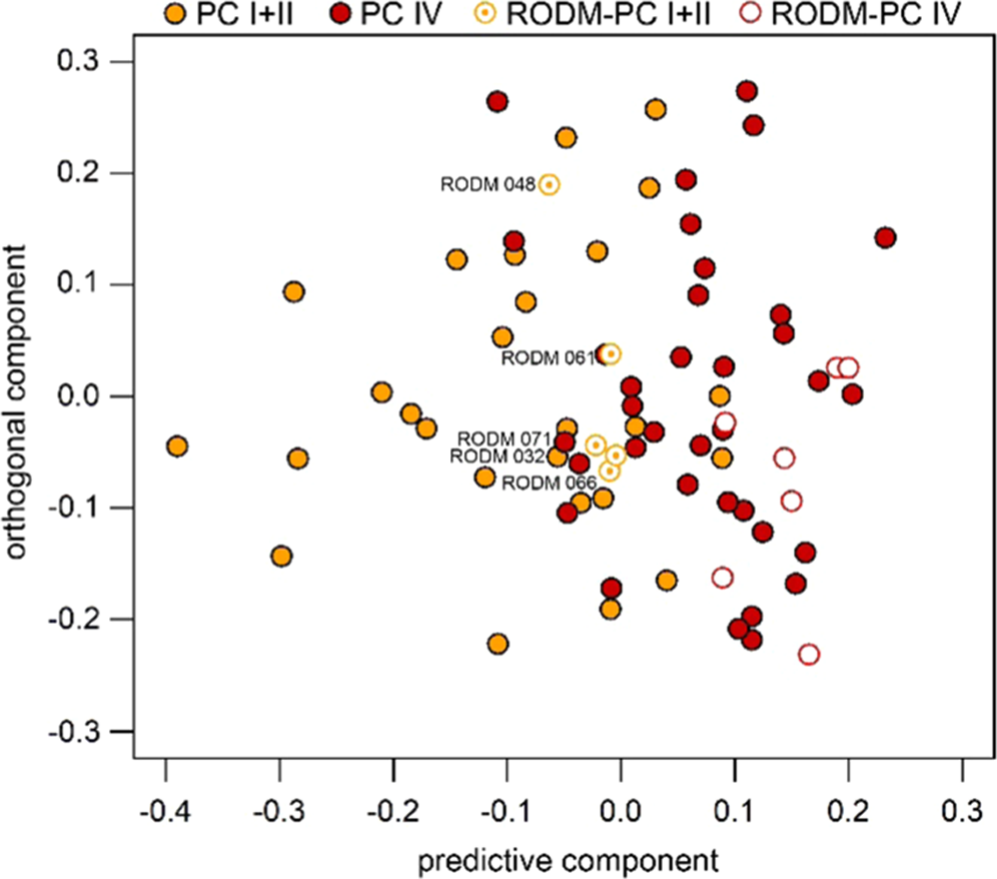
OPLS predictive model based on PC I + II/PC IV discriminant analysis including the classification of 12 marked RODM samples.

The above performed discriminant analyses and the described behavior of RODM samples do not provide a solid clue as to whether the RODM classification of a given sample belongs to early- or late-stage PC. This question can be answered by classification of these samples into a prediction model based on PC I + II/PC IV discriminant analysis. Despite this, OPLS-DA reached only 71.5% accuracy, as mentioned above, and the provided classification of 12 marked RODM samples corresponds reasonably with the tentative clustering provided by discriminant analysis of individual PC stages with DM2 (Figure 6). The PC I + II/PC IV-based predictive model labeled five RODM samples as PC I + II and seven as PC IV (Table 5, last column). The agreement between both approaches reaches 75% as the assignment of three samples out of 12 does not correspond to each other.

In parallel, the current health condition of all RODM patients was reassessed by physicians. Medical data collected to date also suggest that the RODM patients selected by the PC I + II/DM2 predictive model suffer from milder pathological changes in the pancreas than those selected by the PC IV/DM2 model. It is probably more convenient to speak of pathological changes in the pancreas rather than of pancreatic cancer. Nevertheless, two of the patients selected by the model underwent surgical intervention in the meantime, and an early carcinoma was diagnosed from the resected tissue. Some of the patients suffer from other pancreas pathologies, such as severe chronic pancreatitis, focal necrotizing pancreatitis, or intraductal papillary mucinous neoplasm (IPMN). Some of the patients face severe diabetes or other types of cancer. Up to this point, the predictive model provides satisfactory results. However, due to an unspecified number of accumulated uncertainties affecting the accuracy of the predictive model, a number of misclassifications took place. A normal pancreas was found in the patient marked RODM 071, and two other patients underwent pancreas resection in the meantime resulting in early-stage cancer diagnosis. The current health condition of 12 patients marked RODM is summarized in Table 6, and an overview of the whole group can be found in Table S7.

**Table 6:** Current Health Condition of RODM Samples Selected by the Prediction Model

sample	class.	sample col.	DM diag.	birth	current health condition
RODM 001	IV	11/2016	11/2015	1960	post-acute pancreatitis, nonspecific parenchymal changes
RODM 008^a^	III/IV	02/2017	04/2016	1944	severe chronic pancreatitis
RODM 011^a^	IV	03/2017	03/2016	1956	04/2017 resection, early-stage PC
RODM 015^a^	IV	04/2017	04/2016	1950	IPMN
RODM 032	IV	07/2017	05/2017	1946	2021 diag. metastatic renal cancer
RODM 036^a^	IV	07/2017	06/2017	1961	01/2018 resection, chronic pancreatitis with focal necrotic acinar tissue
RODM 037	IV	08/2017	04/2017	1956	polymorbid patient with severe DM
RODM 048	I + II	04/2018	04/2017	1954	05/2018 resection, early-stage PC
RODM 061	I + II	10/2018	10/2016	1954	ulcerative colitis, celiac disease
RODM 065	I + II	11/2018	11/2018	1965	severe chronic pancreatitis
RODM 066	IV	12/2018	12/2015	1957	pancreatic fluid collection with signs of an infection
RODM 071^a^	I + II	03/2019	02/2019	1950	normal pancreas

a Patients identified as at-risk of future development of PC in the previous work (ref (12)).

In summary, the presented predictive model based on discriminant analyses of individual PC stages and the DM2 group is able to identify at-risk individuals recruited from RODM patients. The model based on PC IV/DM2 discrimination selects all at-risk individuals, while the model based on PC I + II/DM2 discrimination selects predominantly patients suffering from mild pathological changes in the pancreas. Further precision of the model can be definitely achieved by better homogeneity of the groups involved. The main difficulty is linked with the proper determination of the PC clinical stage, which can be provided only from a resected tissue by a pathologist. This issue is then reflected in the limited number of early-stage samples, which are rather rare, resulting in improper combination of the samples to obtain a balanced number of samples within each group.

## Conclusions

The OPLS discriminant analysis based on concentration profiles of 61 blood plasma metabolites detected by ^1^H NMR is capable of differentiation between pancreatic cancer (PC) patients from both healthy controls (HCs) and diabetes mellitus (DM2) patients, with high-performance values for each stage of pancreatic cancer. The simultaneous discrimination of PC samples from both control groups can also be achieved using a panel of nine metabolites, which were found of statistical significance in each discriminant analysis. The metabolites with the most altered concentration levels can be attributed to well-known biological processes bound with cancer cell growth and proliferation. Unfortunately, mutual discrimination of individual PC stages was not satisfactory using the current data set. The found changes in concentration levels of the metabolites are rather subtle across PC stages; only the discrimination between early and metastatic stages was achieved with 71.5% accuracy.

The discriminant analyses between individual PC stages and the DM2 group were subsequently used as predictive models for classification of patients with recent-onset diabetes mellitus (RODM) to identify individuals with increased risk of pancreatic cancer development. It was found that the PC I + II/DM2 predictive model selects predominantly patients suffering from mild pathological changes in the pancreas, while the model based on PC IV/DM2 discrimination selects all at-risk individuals. Twelve RODM patients out of 59 were identified as at-risk, and four of them were classified as at moderate risk. These findings were supported by the classification of selected at-risk individuals by PC I + II/PC IV discriminant analysis and by assessment of the current health condition of the whole RODM group. A final comparison of the results shows one RODM sample being classified as false positive and two samples as false negative.

The current predictive model provides quite satisfactory results, and further precision can be reached by increasing the homogeneity of the groups involved. This is mainly associated with the proper determination of the PC clinical stage, providing a balanced number of samples in each group to preclude improper merging. Furthermore, common biological characteristics such as age and sex should be also considered.

## Supporting Information Available

Table S1: statistical significance of biochemical characteristics. Table S2: clinical characteristics of the samples studied. Table S3: list of metabolites assigned. Figure S1: orthogonal partial least squares discriminant analyses. Table S4: statistical significance of metabolite profiles. Table S5: fold-change analysis. Table S6: comparison table of AUC values. Figure S2: box plots of complete set of metabolites. Figure S3: box plots of complete set of metabolites for finer splitting of pancreatic cancer patients. Figure S4: principal component analysis of pancreatic cancer stages. Figure S5: partial least square discriminant analysis of pancreatic cancer stages. Figure S6: principal component analysis of pancreatic cancer stages with graphical correlation with CA 19-9 marker. Table S7: current health condition of all recent-onset diabetes mellitus patients (PDF)

## Abbreviations

AUC: area under the curve
CA 19-9: carbohydrate antigen 19-9
CEA: carcinoembryonic antigen
CI: confidence interval
CPMG: Carr–Purcell–Meiboom–Gill
CT: computed tomography
DM2: type 2 diabetes mellitus (patients)
D_2_O: deuterated water
ERCP: endoscopic retrograde cholangiopancreatography
EUS: endoscopic ultrasound
FPG: fasting plasma glucose
GC/MS: gas chromatography-mass spectrometry
HbA1c: glycated hemoglobin
HC: healthy controls
HSA: human serum albumin
IPMN: intraductal papillary mucinous neoplasm
K_3_EDTA: tri potassium ethylenediaminetetraacetic acid
LC-TQ/MS: liquid chromatography-triple quadrupole mass spectrometry
M: metastasis
MRI: magnetic resonance imaging
MS: mass spectrometry
N: number of attacked lymph nodes
NaN_3_: sodium azide
NMR: nuclear magnetic resonance
OPLS-DA: orthogonal partial least squares-discriminant analysis
PC: pancreatic cancer (patients)
PCA: principal component analysis
ROC: receiver-operating characteristic
RODM: recent-onset diabetes mellitus (patients)
T: tumor
T3cDM: type 3c diabetes mellitus (pancreatogenic diabetes)
TNM: classification of malignant tumors
TSP: sodium salt of trimethylsilyl-[2,2,3,3-*d*_4_]-propionic acid
UICC: Union for International Cancer Control
