# Supplementary material for: Prediction of Pathologic Change Development in the Pancreas Associated with Diabetes Mellitus Assessed by NMR Metabolomics
Source: J Proteome Res. 2023 Apr 5;22(6):1936–46. doi: 10.1021/acs.jproteome.3c00047 (PMC10243102; doi:10.1021/acs.jproteome.3c00047)
Supplement: Supplementary file 1 — pr3c00047_si_001.pdf [file pr3c00047_si_001.pdf]

A Prediction of Pathologic Changes Development in the Pancreas Associated with Diabetes Mellitus Assessed by NMR Metabolomics

Lenka Michálková[1,2,], Štěpán Horník[1], Jan Sýkora[3,*], Vladimír Setnička[2] and Bohuš Bunganič[4,*]*

[1]Institute of Chemical Process Fundamentals of the CAS, Prague 6, Czech Republic

[2]Department of Analytical Chemistry, University of Chemistry and Technology Prague, Prague 6, Czech Republic

[3]Central NMR laboratory, University of Chemistry and Technology Prague, Prague 6, Czech Republic

[4]Department of Internal Medicine, 1st Faculty of Medicine of Charles University and Military University Hospital, Prague 6, Czech Republic

A table of content

Table S1. Statistical significance of biochemical characteristics………………………………… S2

Table S2. Clinical characteristics of the samples studied……………………………………… S3

Table S3. A list of metabolites assigned ……………………………………………..………… S5

Figure S1. Orthogonal partial least squares discriminant analyses………..…………………… S7

Table S4. Statistical significance of metabolite profiles…………………..…………………… S8

Table S5. Fold-change analysis…………………………………………………………………… S10

Table S6. Comparison table of AUC values ……………………………………………………… S12

Figure S2. Box plots of complete set of metabolites …………………………………………… S13

Figure S3. Box plots of complete set of metabolites for finer splitting of pancreatic cancer patients ………………………………………….…………………………………………… S16

Figure S4. Principal component analysis of pancreatic cancer stages ………………………… S19

Figure S5. Partial least square discriminant analysis of pancreatic cancer stages ……………… S19

Figure S6. Principal component analysis of pancreatic cancer stages with graphical correlation with CA 19-9 marker…………………………………………………………………………… S20

Table S7. Current health condition of all recent-onset diabetes mellitus patients……………... S21

Statistical significance of biochemical characteristics

Table S1. Statistical significance of age, sex and BMI in pair discriminations. Statistical significance of the parameter is highlighted in bold.

Discrimination	Age (*P*-value)	Sex (χ^2)	BMI (*P*-value)
HC vs PC I+II	**0.014**	0.61	0.170
HC vs PC III	**0.009**	**6.62**	0.468
HC vs PC IV	**0.036**	1.56	0.492
DM2 vs PC I+II	0.657	0.08	**0.017**
DM2 vs PC III	0.645	2.52	**0.001**
DM2 vs PC IV	0.838	0.01	**<0.001**
HC vs DM2	**0.020**	1.24	**<0.001**
PC I+II vs PC III	0.988	3.20	0.494
PC I+II vs PC IV	0.539	0.17	0.064
PC III vs PC IV	0.523	2.27	0.195

Biochemical characteristics of the samples

Table S2. Clinical characteristics of the samples studied. Data are reported as mean ± standard deviation, or count.

		PC I+II (*n*=26)	PC III (*n*=27)	PC IV (*n*=35)	PC (*n* = 88)	HC (*n* = 28)	T2DM (*n* = 32)	RODM (*n* = 59)
Age		69 ± 9.5	69 ± 8.3	67 ± 9.3	68 ± 9.0	64 ± 9.7	68 ± 9.0	65 ± 8.24
Female		14	8	17	39	19	16	32
Male		12	19	18	49	9	16	27
Urea	$mmol{\cdot}L^{-1}$	4.97 ± 1.47	4.82 ± 1.16	4.98 ± 1.64	4.93 ± 1.47	4.88 ± 1.32	5.90 ± 2.25	5.75 ± 2.04
Creatinine	$\mu mol{\cdot}L^{-1}$	72.06 ± 18.29	285.79 ± 1123.71	68.09 ± 15.02	139.20 ± 637.21	72.38 ± 18.10	82.46 ± 19.43	76.15 ± 16.87
Uric acid	$\mu mol{\cdot}L^{-1}$	289.31 ± 91.74	272.75 ± 84.11	256.76 ± 89.55	271.45 ± 88.57	290.37 ± 66.29	345.93 ± 99.00	331.60 ± 89.15
Sodium	$mmol{\cdot}L^{-1}$	139.87 ± 3.34	140.84 ± 3.71	139.49 ± 2.98	140.01 ± 3.33	141.64 ± 2.03	141.28 ± 1.98	140.82 ± 3.11
Potassium	$mmol{\cdot}L^{-1}$	4.27 ± 0.37	4.41 ± 0.40	4.35 ± 0.57	4.34 ± 0.47	4.46 ± 0.39	4.61 ± 0.48	4.45 ± 0.44
Chlorides	$mmol{\cdot}L^{-1}$	100.92 ± 3.85	102.47 ± 3.49	100.35 ± 3.52	101.15 ± 3.68	102.68 ± 2.15	101.95 ± 3.67	101.53 ± 3.27
Calcium total	$mmol{\cdot}L^{-1}$	2.38 ± 0.16	2.43 ± 0.43	2.31 ± 0.15	2.36 ± 0.27	2.41 ± 0.10	2.46 ± 0.15	2.42 ± 0.11
Calcium ionized	$mmol{\cdot}L^{-1}$	1.24 ± 0.06	1.26 ± 0.11	1.51 ± 1.71	1.35 ± 1.08	1.23 ± 0.05	1.24 ± 0.06	1.23 ± 0.05
Phosphorus	$mmol{\cdot}L^{-1}$	1.20 ± 0.18	1.18 ± 0.20	1.14 ± 0.18	1.17 ± 0.18	1.13 ± 0.16	1.17 ± 0.20	1.11 ± 0.16
Magnesium	$mmol{\cdot}L^{-1}$	0.83 ± 0.09	0.80 ± 0.14	0.84 ± 0.09	0.83 ± 0.11	0.86 ± 0.07	0.81 ± 0.10	0.84 ± 0.08
Bilirubin total	$\mu mol{\cdot}L^{-1}$	50.00 ± 70.72*	39.78 ± 47.94*	58.34 ± 81.39*	50.42 ± 69.49*	10.54 ± 4.37	10.69 ± 5.94	10.59 ± 6.62
Bilirubin direct	$\mu mol{\cdot}L^{-1}$	42.46 ± 63.79*	31.14 ± 43.54*	49.37 ± 72.82*	41.98 ± 62.53*	4.20 ± 1.49	4.64 ± 2.38	4.49 ± 2.36
ALT	$\mu kat{\cdot}L^{-1}$	4.17 ± 13.42	1.60 ± 2.38	1.94 ± 3.32	2.50 ± 7.72	0.47 ± 0.29	0.56 ± 0.32	0.54 ± 0.37
AST	$\mu kat{\cdot}L^{-1}$	1.11 ± 1.70	0.87 ± 0.93	1.69 ± 3.65	1.28 ± 2.56	0.41 ± 0.18	0.47 ± 0.25	0.43 ± 0.21
GMT	$\mu kat{\cdot}L^{-1}$	6.73 ± 10.52	8.20 ± 12.41	5.71 ± 6.55	6.76 ± 9.75	0.75 ± 1.53	1.66 ± 2.59	0.92 ± 1.42
ALP	$\mu kat{\cdot}L^{-1}$	3.40 ± 2.82	4.91 ± 7.42	5.69 ± 5.31	4.77 ± 5.51*	1.48 ± 1.06	1.40 ± 0.61	1.59 ± 1.52
Amylase total	$\mu kat{\cdot}L^{-1}$	0.86 ± 0.50	0.80 ± 0.78	0.81 ± 0.61	0.82 ± 0.63	1.60 ± 2.69	1.09 ± 0.57	0.97 ± 0.48
Pancreatic amylase	$\mu kat{\cdot}L^{-1}$	0.42 ± 0.45	0.42 ± 0.76	0.44 ± 0.57	0.43 ± 0.60	0.93 ± 2.55	0.58 ± 0.42	0.42 ± 0.26
Total cholesterol	$mmol{\cdot}L^{-1}$	4.77 ± 1.81	4.68 ± 2.14	4.81 ± 1.35	4.76 ± 1.75	5.57 ± 0.93	4.25 ± 1.17	4.95 ± 1.19
LDL	$mmol{\cdot}L^{-1}$	2.45 ± 0.81	2.79 ± 1.80	3.00 ± 1.29	2.78 ± 1.39	3.26 ± 1.02	2.82 ± 3.55	2.73 ± 1.02
HDL	$mmol{\cdot}L^{-1}$	0.99 ± 0.45	1.04 ± 0.45	0.98 ± 0.50	1.00 ± 0.47	1.96 ± 1.32	1.29 ± 0.34	1.37 ± 0.54

		PC I+II (*n*=26)	PC III (*n*=27)	PC IV (*n*=35)	PC (*n* = 88)	HC (*n* = 28)	T2DM (*n* = 32)	RODM (*n* = 59)
Triglycerides	mmol·L^{-1}	1.44 ± 0.58	1.41 ± 0.75	1.50 ± 0.58	1.45 ± 0.64	1.27 ± 0.52	1.72 ± 0.96	1.79 ± 1.10
FPG	mmol·L^{-1}	7.15 ± 2.90	6.45 ± 2.54	7.42 ± 3.36	7.03 ± 2.98	5.44 ± 0.52	7.90 ± 1.81	7.10 ± 2.57
C-peptide	pmol·L^{-1}	822.95 ± 384.91	590.03 ± 339.50	617.75 ± 427.14	669.62 ± 397.94	710.45 ± 325.94	1065.67 ± 337.73	1023.92 ± 591.36
Albumin	g·L^{-1}	37.90 ± 5.46	38.52 ± 5.61	37.42 ± 6.41	37.91 ± 5.84	45.44 ± 3.80	44.66 ± 5.31	44.47 ± 4.34
Total protein	g·L^{-1}	65.05 ± 7.42	63.30 ± 8.12	63.86 ± 8.76	64.04 ± 8.12	70.04 ± 5.92	71.81 ± 7.62	69.49 ± 6.09
Prealbumin	g·L^{-1}	0.20 ± 0.07	0.23 ± 0.14	0.17 ± 0.08	0.20 ± 0.10	0.27 ± 0.07	0.30 ± 0.07	0.28 ± 0.08
CRP	mg·L^{-1}	15.81 ± 32.12	10.69 ± 15.48	75.65 ± 285.05	38.04 ± 181.85	2.93 ± 4.05	16.31 ± 71.56	7.39 ± 17.35
CA 19-9	kU·L^{-1}	564.64 ± 972.76	1553.00 ± 3268.84	13339.05 ± 48104.00	5999.14 ± 30903.84	9.99 ± 5.45	18.64 ± 14.70	15.26 ± 11.73
CEA	kU·L^{-1}	5.18 ± 7.09	7.29 ± 11,23	79.77 ± 256.00	36.89 ± 161.01	2.29 ± 1.56	2.45 ± 1.55	2.32 ± 2.11
HbA1c	mmol·L^{-1}	52.54 ± 18.96	50.91 ± 13.91	51.26 ± 21.72	51.57 ± 18.81	36.22 ± 3.59	52.19 ± 15.24	49.37 ± 17.26
Leukocytes	10^9·L^{-1}	7.39 ± 2.27	7.08 ± 2.50	8.14 ± 2.42	7.60 ± 2.42	6.52 ± 2.09	7.57 ± 1.98	7.32 ± 2.18
Erythrocytes	10^{12}·L^{-1}	4.21 ± 0.49	4.39 ± 0.61	5.03 ± 4.67	4.59 ± 3.01	4.73 ± 0.43	4.79 ± 0.72	4.86 ± 0.51
Haemoglobin	g·L^{-1}	123.54 ± 16.13	132.04 ± 17.60	122.29 ± 16.95	125.50 ± 17.23	141.89 ± 14.25	143.47 ± 19.30	143.40 ± 15.51
Haematocrit	g·L^{-1}	1.55 ± 6.01	0.39 ± 0.05	0.36 ± 0.05	0.73 ± 3.30	0.42 ± 0.04	0.43 ± 0.05	0.42 ± 0.04
Thrombocytes	10^9·L^{-1}	244.23 ± 67.35	238.20 ± 135.93	234.14 ± 80.61	238.37 ± 95.75	239.44 ± 56.64	228.44 ± 73.46	229.33 ± 79.24
INR		1.06 ± 0.12	1.10 ± 0.30	1.15 ± 0.21	1.110 ± 0.22	0.99 ± 0.05	1.06 ± 0.15	0.97 ± 0.15
Aptt		25.14 ± 5.92	27.86 ± 10.29	26.14 ± 5.83	26.33 ± 7.41	27.36 ± 5.53	26.78 ± 8.99	25.57 ± 8.34

* Blood was collected before ERCP drain (age), which caused hyperbilirubinemia. (Median of total bilirubin 11.7 µmol·L^{-1}, median of direct bilirubin 5.1 µmol·L^{-1}, ALP median 1.57 µkat·L^{-1}).

Abbreviation: ALP, alkaline phosphatase; ALT, alanine transaminase; Aptt, activated partial thromboplastin time; AST, aspartate transaminase; CA 19-9, carbohydrate antigen 19-9; CEA, carcinoembryonic antigen; CRP, C-reactive protein; ERCP, endoscopic retrograde cholandiopancreatography; FPG, fasting plasma glucose; GMT, gamma-glutamyl transferase; HBA_{1c}, glycated haemoglobin; HDL, high density lipoproteins; INR, international normalised ration; LDL, low density lipoproteins.

Metabolite identification

Table S3. Complete list of metabolites assigned

Metabolite	Signal ppm (multiplicity)													
2-Hydroxybutyrate	3.99 (t)	1.73 (m)	1.64 (m)	**0.89 (t)**										
2-Hydroxyisobutyrate	**1.34 (s)**													
2-Hydroxyisovalerate	3.84 (d)	2.00 (m)	0.95 (d)	**0.82 (d)**										
2-Oxoglutarate	**2.99 (t)**	2.43 (t)												
2-Oxoisocaproate	**2.60 (d)**	2.08 (m)	**0.93 (d)**											
3-Hydroxybutyrate	4.14 (m)	2.40 (m)	2.29 (m)	**1.19 (d)**										
3-Hydroxyisobutyrate	3.69 (q)	3.53 (q)	2.48 (m)	**1.06 (d)**										
3-Hydroxyisovalerate	2.35 (s)	**1.26 (s)**												
3-Methyl-2-oxovalerate	2.92 (m)	1.69 (m)	1.45 (m)	**1.09 (d)**	**0.88 (t)**									
Acetate	**1.91 (s)**													
Acetoacetate	3.43 (s)	**2.27 (s)**												
Acetone	**2.22 (s)**													
Alanine	3.77 (q)	**1.47 (d)**												
Arginine	3.76 (t)	3.24 (t)	1.92 (m)	1.88 (m)	**1.72 (m)**	**1.64 (m)**								
Asparagine	3.98 (q)	**2.93 (dd)**	**2.83 (dd)**											
Betaine	3.89 (s)	**3.25 (s)**												
Carnitine	4.56 (m)	3.43 (m)	3.40 (m)	3.22 (s)	**2.45 (dd)**	**2.41 (dd)**								
Choline	**4.05 (m)**	3.51 (dd)	**3.19 (s)**											
Citrate	**2.68 (d)**	2.52 (d)												
Creatine	3.92 (s)	**3.02 (s)**												
Creatinine	4.04 (s)	**3.03 (s)**												
Dimethylamine	**2.71 (s)**													
Ethanol	3.65 (q)	**1.17 (t)**												
Formate	**8.44 (s)**													
Fumarate	**6.51 (s)**													
Gluconate	4.12 (d)	**4.02 (t)**	3.82 (m)	3.78 (m)	3.76 (m)	3.66 (m)								
Glucose	**5.24 (d)**	4.64 (d)	3.90 (dd)	3.84 (m)	3.82 (m)	3.75 (dd)	3.71 (dd)	3.70 (dd)	3.52 (dd)	3.48 (t)	3.46 (m)	3.40 (t)	3.39 (m)	3.23 (dd)
Glutamate	3.75 (dd)	**2.36 (m)**	**2.32 (m)**	2.12 (m)	2.04 (m)									
Glutamine	3.75 (t)	**2.46 (m)**	**2.42 (m)**	2.14 (m)	2.10 (m)									
Glycine	**3.55 (s)**													
Glycolate	**3.93 (s)**													
Histidine	**7.78 (d)**	7.05 (d)	3.97 (dd)	3.22 (dq)	3.11 (dd)									
Hydroxyacetone	4.37 (s)	2.15 (s)												
Hypoxanthine	**8.19 (s)**	**8.18 (s)**												

Metabolite	Signal ppm (multiplicity)													
Isoleucine	3.66 (d)	1.97 (m)	1.46 (m)	1.25 (m)	**1.00 (d)**	**0.93 (t)**								
Isopropanol	4.01 (m)	**1.16 (d)**												
Lactate	4.10 (q)	**1.32 (d)**												
Leucine	3.72 (m)	1.74 (m)	1.70 (m)	1.67 (m)	**0.96 (d)**	**0.94 (d)**								
Lysine	3.74 (t)	**3.02 (t)**	1.91 (m)	1.87 (m)	1.72 (m)	1.50 (m)	1.43 (m)							
Mannose	**5.17 (d)**	4.89 (d)	3.94 (dd)	3.92 (dd)	3.90 (dd)	3.86 (dd)	3.84 (dd)	3.81 (m)	3.76 (dd)	3.73 (dd)	3.65 (t)	3.65 (dd)	3.57 (t)	3.37 (m)
Methanol	3.35 (s)													
Methionine	3.84 (dd)	**2.63 (t)**	2.18 (m)	**2.13 (s)**	2.10 (m)									
N,N-Dimethylglycine	3.71 (s)	**2.91 (s)**												
N-Acetylcysteine	4.36 (m)	2.92 (dd)	2.89 (dd)	**2.06 (s)**										
N-Acetylglycine	3.74 (d)	**2.03 (s)**												
N-Phenylacetylglycine	7.41 (m)	**7.34 (m)**	**7.34 (m)**	3.74 (d)	3.67 (s)									
Ornithine	3.77 (t)	**3.04 (t)**	1.93 (m)	1.82 (m)	1.73 (m)									
Phenylalanine	**7.42 (t)**	**7.36 (m)**	**7.32 (d)**	3.98 (q)	3.27 (q)	3.11 (q)								
Proline	4.12 (dd)	3.41 (dt)	3.33 (dt)	2.34 (m)	**2.06 (m)**	**2.03 (m)**	**1.98 (m)**							
Propionate	2.17 (q)	**1.04 (t)**												
Pyruvate	**2.36 (s)**													
Serine	**3.98 (dd)**	**3.93 (dd)**	3.82 (dd)											
Succinate	**2.39 (s)**													
Threonine	**4.24 (m)**	3.57 (d)	1.32 (d)											
Tryptophan	**7.73 (d)**	**7.53 (d)**	7.31 (s)	**7.27 (m)**	7.19 (m)	4.05 (dd)	3.47 (dd)	3.29 (dd)						
Tyrosine	**7.18 (d)**	**6.89 (d)**	3.93 (dd)	3.18 (dd)	3.04 (dd)									
Urea	**5.77 (s)**													
Uridine	**7.86 (d)**	**5.90 (d)**	**5.89 (d)**	4.34 (dd)	4.22 (dd)	4.12 (m)	3.90 (dd)	3.80 (dd)						
Valine	3.60 (d)	2.26 (m)	**1.03 (d)**	**0.98 (d)**										
myo-Inositol	**4.05 (t)**	3.61 (t)	3.53 (dd)	3.27 (t)										
Methylhistidine	7.61 (s)	**6.98 (s)**	3.95 (dd)	3.67 (s)	3.14 (dd)	3.04 (dd)								

The signals used for initial compound fitting are in bold. Signal multiplicity: singlet (s), doublet (d), triplet (t), doublet of doublets (dd), doublet of triplets (dt), multiplet (m).

Discriminant analyses of pancreatic cancer stages and both control groups

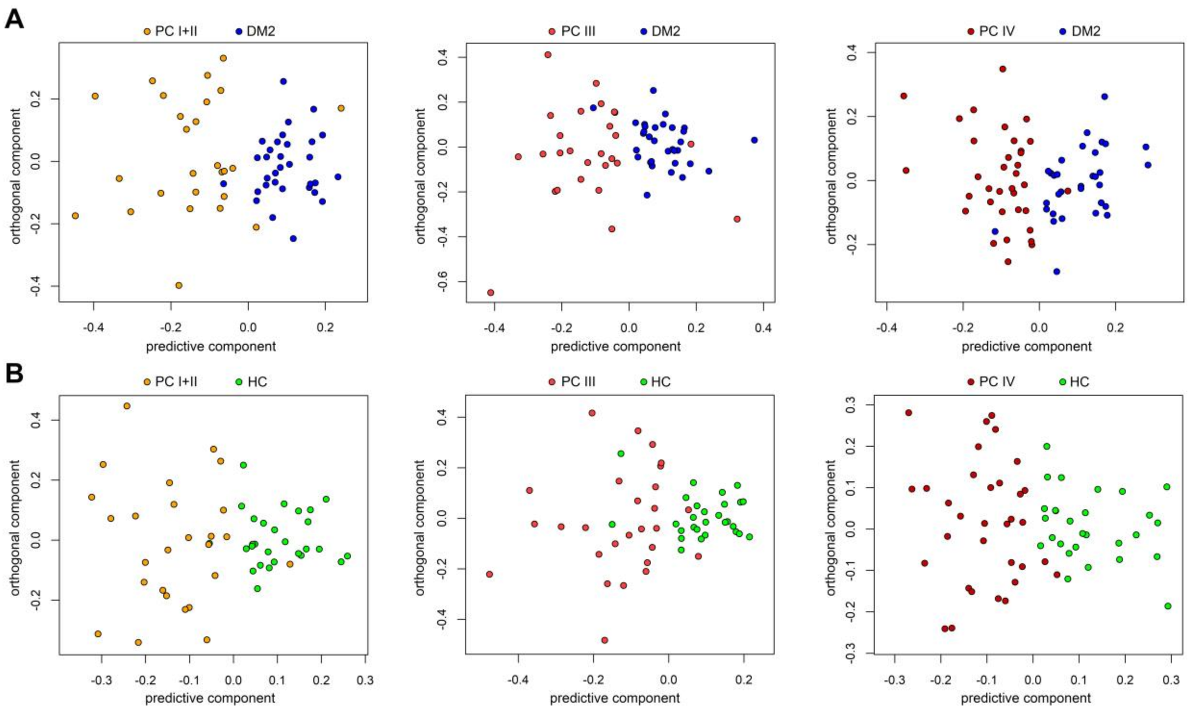

Figure S1. Orthogonal partial least squares discriminant analyses of pancreatic cancer stages (PC I+II, PC III, PC IV) and diabetes mellitus patients (A) and healthy controls (B).

Statistical significance of complete metabolite profiles

Table S4. Statistical significance of metabolite profiles between pancreatic cancer stages (PC I+II, PC III, PC IV) and both control groups (HC, DM2), ***P*-value <0.05**

**Metabolite**	*P*-value					
	HC			DM2		
	PC I+II	PC III	PC IV	PC I+II	PC III	PC IV
2-Hydroxybutyrate	0.1598	**0.0097**	**0.0017**	0.4374	0.1211	**0.0321**
2-Hydroxyisobutyrate	**0.0019**	**0.0180**	**0.0192**	0.1388	0.4561	0.5346
2-Hydroxyisovalerate	0.2316	0.0857	**0.0010**	0.3955	0.3668	**0.0321**
2-Oxoglutarate	0.5032	0.7567	0.8962	0.6956	0.3236	0.1696
2-Oxoisocaproate	0.5688	0.9036	0.8673	0.7576	0.8015	0.8054
3-Hydroxybutyrate	**0.0085**	**0.0008**	**0.0001**	0.0950	**0.0304**	**0.0030**
3-Hydroxyisobutyrate	0.7488	0.4097	0.8962	0.6956	0.3952	0.9062
3-Hydroxyisovalerate	**0.0211**	**0.0377**	**0.0190**	**0.0231**	**0.0452**	**0.0187**
3-Methyl-2-oxovalerate	**0.0353**	**0.0134**	**0.0030**	0.1388	0.0730	**0.0296**
Acetate	0.1598	**0.0344**	**0.0083**	0.9320	0.5661	0.4033
Acetoacetate	**0.0456**	**0.0377**	**0.0001**	0.2599	0.2362	**0.0031**
Acetone	**0.0497**	**0.0420**	0.0998	0.0924	0.0810	0.1838
Alanine	0.1401	0.1352	**0.0002**	0.1651	0.2362	**0.0021**
Arginine	0.1401	0.0857	0.2822	0.1959	0.6586	0.1150
Asparagine	**0.0002**	**0.0000**	**0.0082**	**0.0059**	**0.0008**	0.3263
Betaine	**0.0285**	0.1021	**0.0094**	0.8193	0.6473	0.9262
Carnitine	**0.0115**	**0.0016**	**0.0002**	0.9320	0.3952	0.3263
Choline	0.8303	0.5834	0.3643	0.1388	0.6120	0.5346
Citrate	0.0641	**0.0100**	**0.0095**	0.6956	0.6120	0.5295
Creatine	**0.0001**	**0.0001**	**0.0001**	**0.0003**	**0.0006**	**0.0004**
Creatinine	0.2757	**0.0203**	**0.0080**	0.8392	0.4457	0.3322
Dimethylamine	0.7488	0.1198	0.2423	0.8081	0.2098	0.2377
Ethanol	0.9795	0.0729	**0.0019**	0.9254	0.2098	**0.0064**
Formate	0.2491	**0.0366**	**0.0080**	0.6956	0.5661	0.2121
Fumarate	**0.0026**	**0.0014**	**0.0005**	**0.0122**	**0.0160**	**0.0052**
Gluconate	**0.0285**	**0.0039**	**0.0003**	**0.0003**	**0.0000**	**0.0000**
Glucose	0.0605	0.1021	0.2815	**0.0059**	**0.0304**	**0.0242**
Glutamate	**0.0497**	0.2666	0.0792	0.1598	0.3952	0.1838
Glutamine	**0.0000**	**0.0000**	**0.0000**	0.1019	0.3236	0.2704
Glycine	**0.0005**	**0.0001**	**0.0004**	0.2603	0.2098	0.3263
Glycolate	0.5014	0.8105	0.8962	**0.0173**	0.3302	0.1241
Histidine	**0.0000**	**0.0000**	**0.0000**	0.0679	0.3302	0.1508
Hydroxyacetone	0.5901	0.0526	**0.0019**	0.5223	0.0645	**0.0014**
Hypoxanthine	**0.0007**	0.1021	0.5289	**0.0059**	0.2098	0.5385
Isoleucine	0.1272	0.3371	0.2180	0.4384	0.6942	0.6394
Isopropanol	**0.0214**	**0.0005**	**0.0031**	0.8392	0.3236	0.5041
Lactate	**0.0001**	**0.0008**	**0.0089**	**0.0059**	**0.0304**	0.1532
Leucine	0.6137	1.0000	0.8962	0.6956	0.9494	0.9062
Lysine	**0.0000**	**0.0000**	**0.0004**	**0.0059**	**0.0055**	**0.0269**

**Metabolite**	*P*-value					
	HC			DM2		
	PC I+II	PC III	PC IV	PC I+II	PC III	PC IV
Mannose	**0.0000**	**0.0001**	**0.0000**	**0.0004**	**0.0041**	**0.0000**
Methanol	**0.0123**	**0.0014**	**0.0117**	0.3960	0.1563	0.6760
Methionine	**0.0013**	**0.0001**	**0.0004**	0.1651	0.1421	0.2954
N,N-Dimethylglycine	0.4033	0.9423	0.8867	0.4378	1.0000	0.9111
N-Acetylcysteine	**0.0047**	**0.0007**	**0.0009**	**0.0428**	**0.0123**	**0.0204**
N-Acetylglycine	0.6409	0.0869	0.8962	0.1711	0.5325	0.0649
N-Phenylacetylglycine	0.5673	0.8641	0.7314	0.1388	0.4457	0.4033
Ornithine	**0.0084**	**0.0005**	**0.0115**	0.3751	0.7909	0.2275
Phenylalanine	0.1033	**0.0038**	0.0659	0.3754	0.8596	0.6032
Proline	**0.0187**	**0.0100**	**0.0313**	**0.0059**	**0.0032**	**0.0187**
Propionate	**0.0147**	**0.0279**	**0.0089**	**0.0004**	**0.0032**	**0.0000**
Pyruvate	**0.0018**	**0.0160**	**0.0005**	0.0679	0.2090	**0.0269**
Serine	0.1992	0.2417	**0.0082**	0.1893	0.3302	0.4033
Succinate	**0.0148**	0.0857	0.1037	0.2845	0.7424	0.9751
Threonine	0.4875	0.5846	0.2965	0.1711	0.3302	0.2422
Tryptophan	0.5705	0.6769	0.0665	0.1093	0.0974	0.3836
Tyrosine	**0.0001**	**0.0001**	**0.0002**	0.0760	0.1660	0.2121
Urea	0.7494	0.6374	0.4848	0.8392	0.9494	0.9712
Uridine	**0.0484**	**0.0011**	**0.0001**	0.2228	0.4252	0.6032
Valine	0.4274	0.3973	**0.0444**	0.7576	0.8943	0.1838
myo-Inositol	0.5722	0.6364	0.8962	0.3102	0.8596	0.8219
Methylhistidine	0.2052	0.2306	0.3639	0.1363	0.1421	0.2696

Table S5. Fold change of metabolite profiles between pancreatic cancer stages (PC I+II, PC III, PC IV) and both control groups (HC, DM2). Fold change values were calculated as the ratio of median of PC stage to median of control group, expressed as logarithm of base 2.

Metabolite	log$_2$(Fold Change)					
	HC			DM2		
	PC I+II	PC III	PC IV	PC I+II	PC III	PC IV
2-Hydroxybutyrate	0.827	0.481	0.760	0.742	0.397	0.675
2-Hydroxyisobutyrate	0.662	0.473	0.450	0.518	0.328	0.305
2-Hydroxyisovalerate	0.075	0.242	0.417	0.249	0.416	0.591
2-Oxoglutarate	-0.083	0.018	0.034	0.106	0.206	0.223
2-Oxoisocaproate	-0.144	0.032	-0.013	-0.176	0.001	-0.044
3-Hydroxybutyrate	1.886	1.866	2.304	1.492	1.471	1.910
3-Hydroxyisobutyrate	0.081	0.270	0.147	0.108	0.298	0.175
3-Hydroxyisovalerate	0.437	0.334	0.381	0.459	0.355	0.402
3-Methyl-2-oxovalerate	0.252	0.389	0.421	0.229	0.366	0.398
Acetate	-0.474	-0.562	-0.575	-0.111	-0.200	-0.213
Acetoacetate	1.638	1.364	2.106	0.969	0.695	1.436
Acetone	1.435	1.303	1.049	1.158	1.027	0.773
Alanine	-0.125	-0.138	-0.525	-0.240	-0.253	-0.640
Arginine	-0.184	-0.288	-0.055	0.329	0.225	0.458
Asparagine	-0.622	-0.617	-0.393	-0.451	-0.446	-0.222
Betaine	-0.403	-0.301	-0.321	-0.018	0.084	0.064
Carnitine	-0.357	-0.550	-0.397	-0.061	-0.253	-0.100
Choline	-0.200	-0.121	-0.200	0.189	0.269	0.189
Citrate	-0.437	-0.413	-0.473	-0.155	-0.132	-0.192
Creatine	-1.070	-1.191	-0.975	-1.017	-1.138	-0.923
Creatinine	-0.201	-0.307	-0.356	-0.022	-0.129	-0.177
Dimethylamine	0.082	-0.254	-0.287	-0.027	-0.363	-0.396
Ethanol	-0.015	-0.688	-1.034	0.408	-0.265	-0.610
Formate	-0.460	-0.754	-1.007	0.085	-0.210	-0.463
Fumarate	-1.019	-0.975	-0.845	-0.931	-0.886	-0.757
Gluconate	1.450	1.455	1.714	3.035	3.040	3.299
Glucose	-0.111	-0.268	-0.064	-0.231	-0.389	-0.184
Glutamate	0.345	0.233	0.251	0.376	0.264	0.282
Glutamine	-0.608	-0.572	-0.549	-0.144	-0.107	-0.084
Glycine	-0.586	-0.639	-0.421	-0.259	-0.312	-0.094
Glycolate	0.078	-0.145	-0.024	0.435	0.212	0.333
Histidine	-0.620	-0.528	-0.701	-0.197	-0.105	-0.278
Hydroxyacetone	0.000	0.000	0.000	0.000	0.000	0.000
Hypoxanthine	0.890	0.537	0.085	0.845	0.492	0.040
Isoleucine	0.191	0.129	0.229	0.044	-0.019	0.081
Isopropanol	-0.649	-0.871	-0.718	-0.157	-0.380	-0.227
Lactate	0.535	0.592	0.402	0.486	0.542	0.353
Leucine	0.105	0.150	0.119	0.006	0.050	0.020
Lysine	-0.442	-0.485	-0.519	-0.258	-0.301	-0.334
Mannose	0.746	0.613	0.761	0.620	0.487	0.635

Metabolite	log$_2$(Fold Change)					
	HC			DM2		
	PC I+II	PC III	PC IV	PC I+II	PC III	PC IV
Methanol	-0.778	-1.007	-0.486	-0.236	-0.466	0.055
Methionine	-0.394	-0.468	-0.427	-0.171	-0.245	-0.204
N-N-Dimethylglycine	0.085	0.010	-0.073	-0.048	-0.123	-0.207
N-Acetylcysteine	-1.052	-1.546	-1.127	-0.681	-1.176	-0.757
N-Acetylglycine	-0.254	-0.448	-0.072	0.320	0.125	0.501
N-Phenylacetylglycine	0.129	0.023	0.024	0.350	0.244	0.245
Ornithine	-0.405	-0.516	-0.430	0.195	0.084	0.170
Phenylalanine	-0.165	-0.165	-0.158	0.036	0.036	0.043
Proline	-0.365	-0.387	-0.358	-0.295	-0.318	-0.289
Propionate	0.803	0.834	0.734	1.094	1.125	1.025
Pyruvate	-0.706	-0.768	-0.510	-0.767	-0.830	-0.571
Serine	-0.123	-0.333	-0.320	0.320	0.109	0.122
Succinate	-0.596	-0.360	-0.437	-0.228	0.008	-0.069
Threonine	-0.074	-0.048	-0.182	0.252	0.279	0.145
Tryptophan	-0.133	-0.126	-0.253	0.328	0.334	0.207
Tyrosine	-0.628	-0.625	-0.526	-0.255	-0.252	-0.153
Urea	0.059	0.024	0.053	0.089	0.054	0.082
Uridine	-0.285	-0.418	-0.437	0.299	0.166	0.148
Valine	-0.178	-0.194	-0.301	-0.080	-0.095	-0.202
myo-Inositol	0.144	-0.166	0.011	0.278	-0.031	0.146
Methylhistidine	-1.099	-1.016	-0.470	-0.940	-0.857	-0.311

Performance analyses

Table S6. Comparison table of AUC values for complete metabolic profile and selected metabolites panels.

	AUC					
	HC			DM2		
Metabolites	61	9	8	61	9	8
PC I+II	0.931	0.909	0.925	0.892	0.905	0.908
PC III	0.954	0.922	0.913	0.889	0.931	0.834
PC IV	0.966	0.964	0.925	0.945	0.954	0.888

Univariate statistical analyses

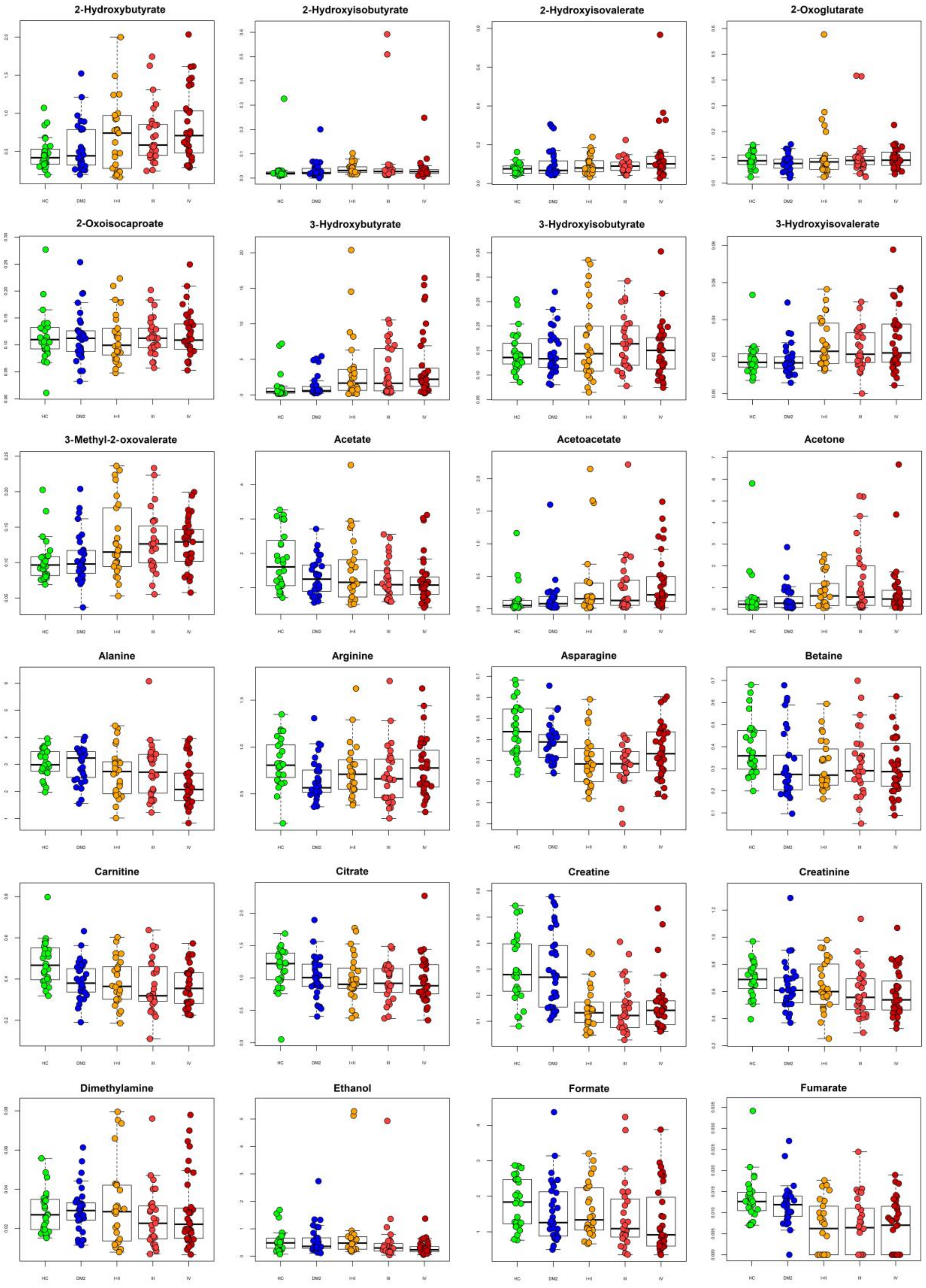

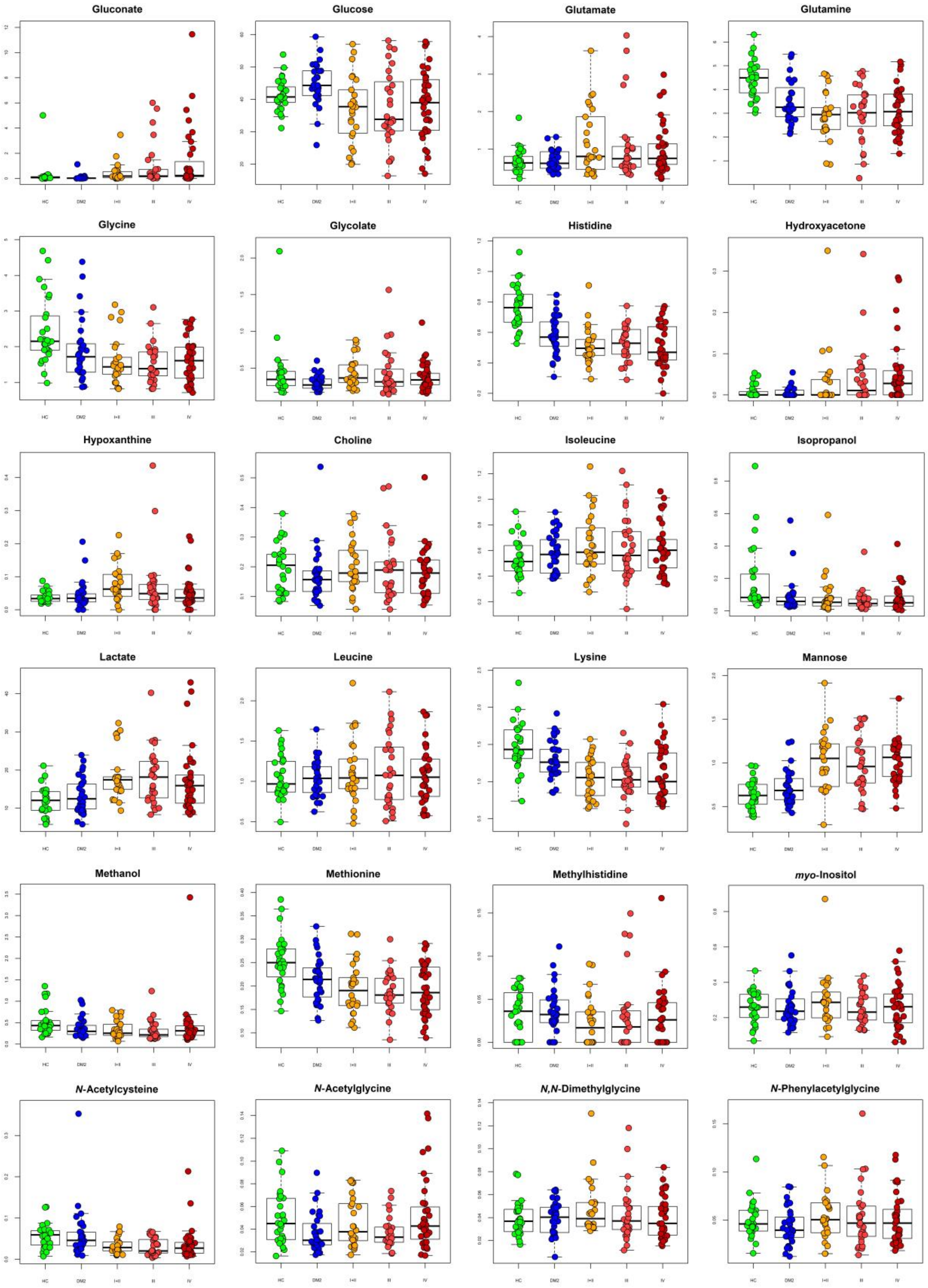
Gluconate
Glucose
Glutamate
Glutamine
Glycine
Glycolate
Histidine
Hydroxyacetone
Hypoxanthine
Choline
Isoleucine
Isopropanol
Lactate
Leucine
Lysine
Mannose
Methanol
Methionine
Methylhistidine
myo-Inositol
N-Acetylcysteine
N-Acetylglycine
N,N-Dimethylglycine
N-Phenylacetylglycine
HC
DM2
I+II
III
IV

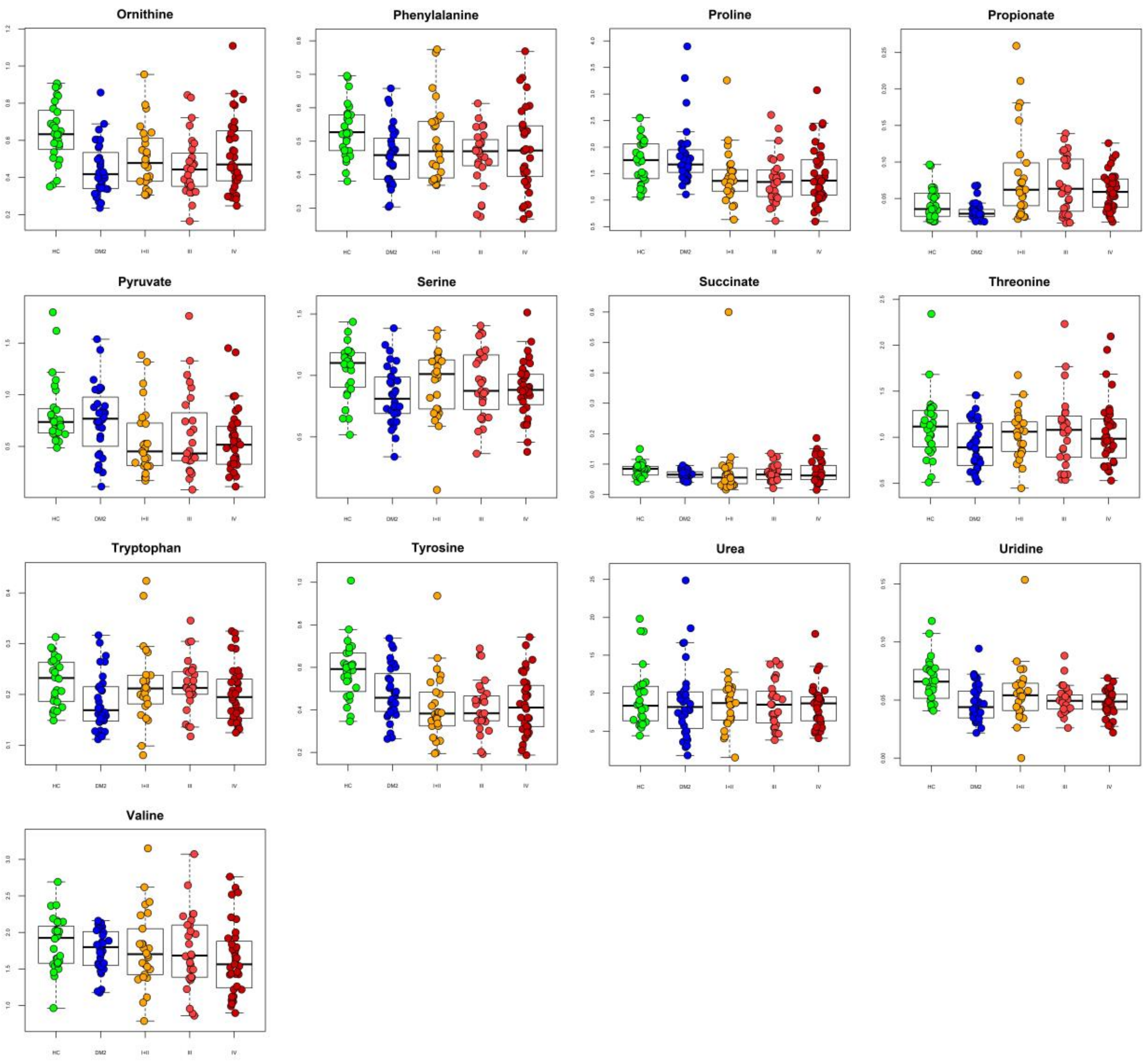

Figure S2. Box plots of complete set of metabolites for healthy controls (HC), diabetes mellitus type 2 patients (DM2), and pancreatic cancer stages (PC I+II, PC III, PC IV).

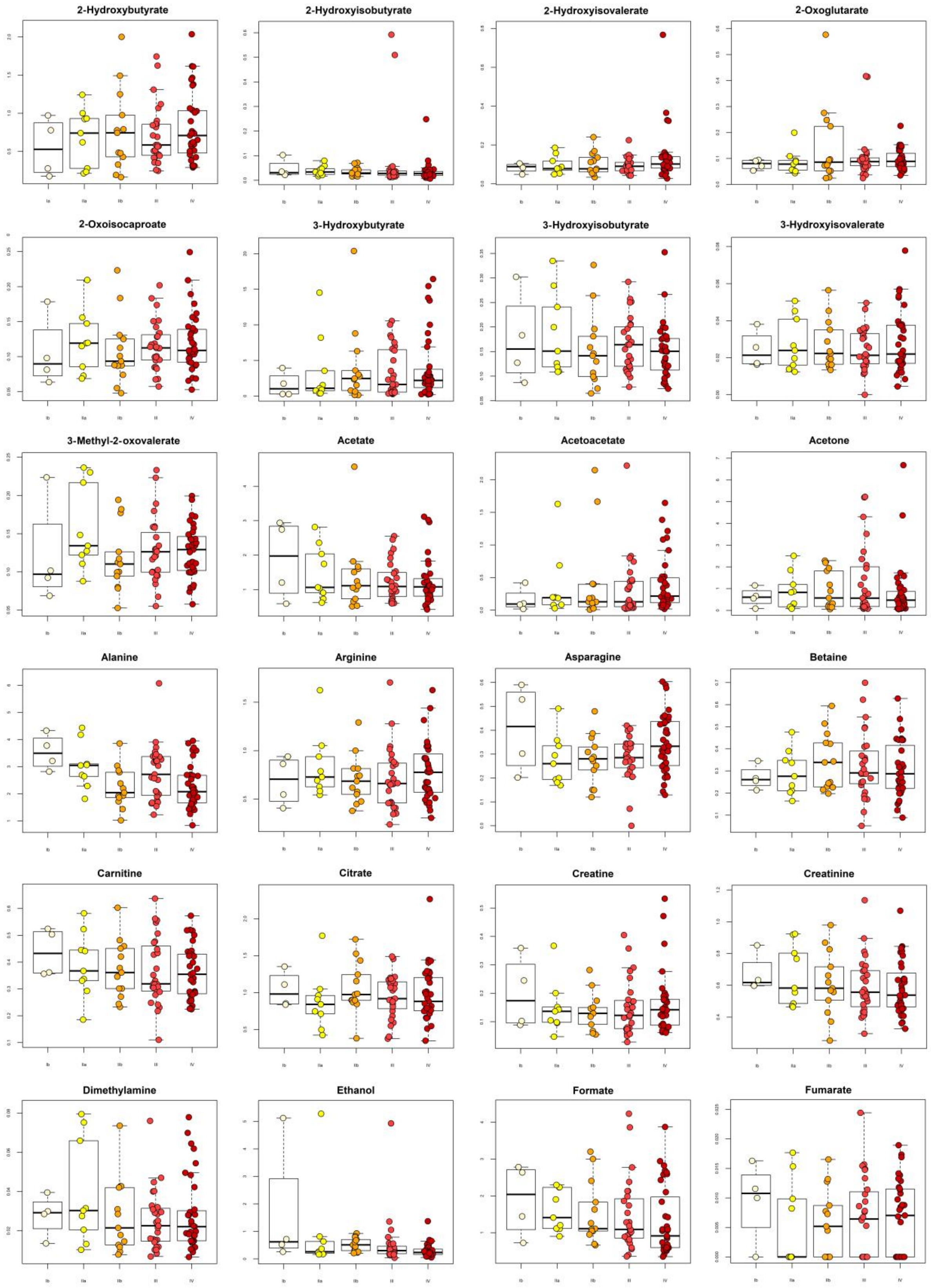
2-Hydroxybutyrate
2-Hydroxyisobutyrate
2-Hydroxyisovalerate
2-Oxoglutarate
2-Oxoisocaproate
3-Hydroxybutyrate
3-Hydroxyisobutyrate
3-Hydroxyisovalerate
3-Methyl-2-oxovalerate
Acetate
Acetoacetate
Acetone
Alanine
Arginine
Asparagine
Betaine
Carnitine
Citrate
Creatine
Creatinine
Dimethylamine
Ethanol
Formate
Fumarate

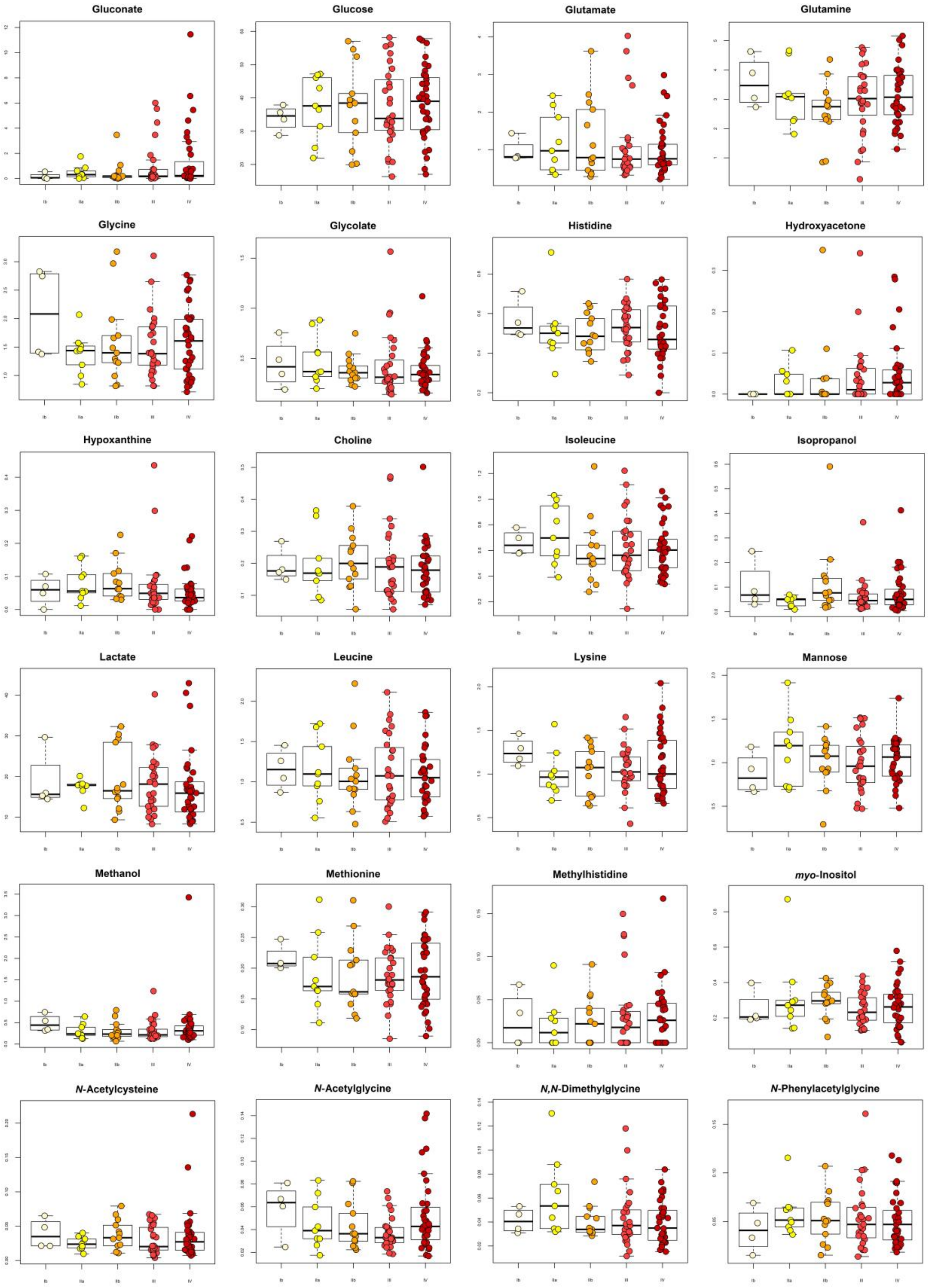
Gluconate
Glucose
Glutamate
Glutamine
Glycine
Glycolate
Histidine
Hydroxyacetone
Hypoxanthine
Choline
Isoleucine
Isopropanol
Lactate
Leucine
Lysine
Mannose
Methanol
Methionine
Methylhistidine
myo-Inositol
N-Acetylcysteine
N-Acetylglycine
N,N-Dimethylglycine
N-Phenylacetylglycine

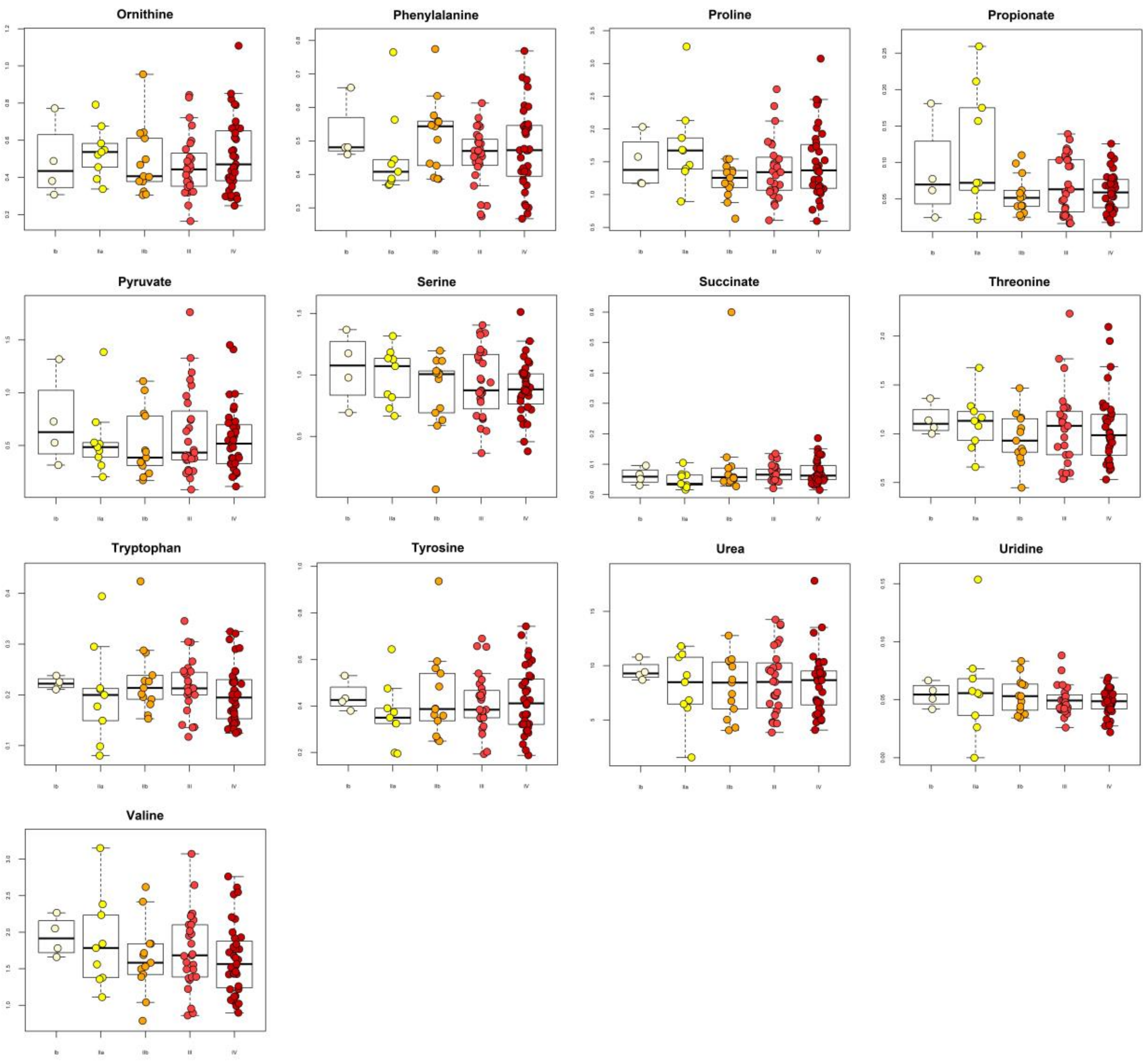

Figure S3. Box plots of complete set of metabolites for finer splitting of pancreatic cancer patients according to TNM classification (Ib, IIa, IIb, III, IV).

Multivariate analyses of pancreatic cancer stages

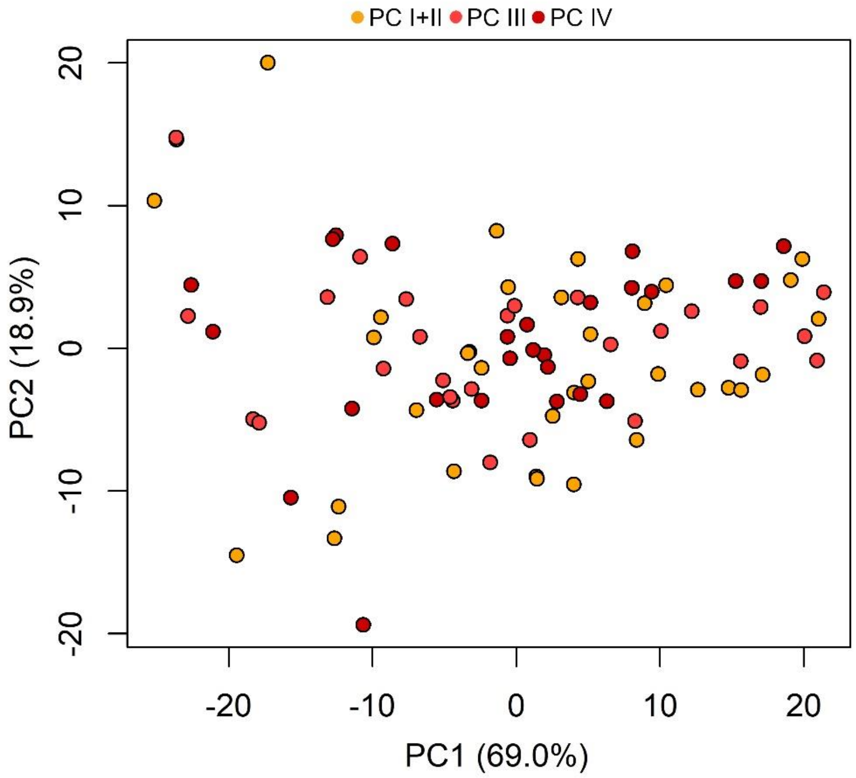

Figure S4. Principal component analysis of pancreatic cancer stages (PC I+II, PC III, PC IV).

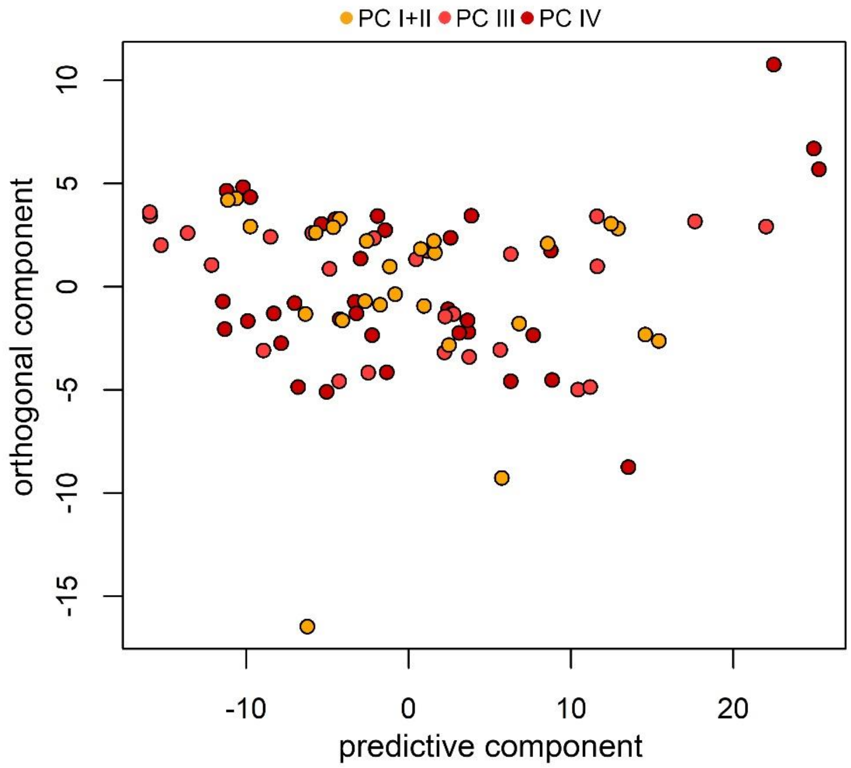

Figure S5. Partial least square discriminant analysis of pancreatic cancer stages (PC I+II, PC III, PC IV).

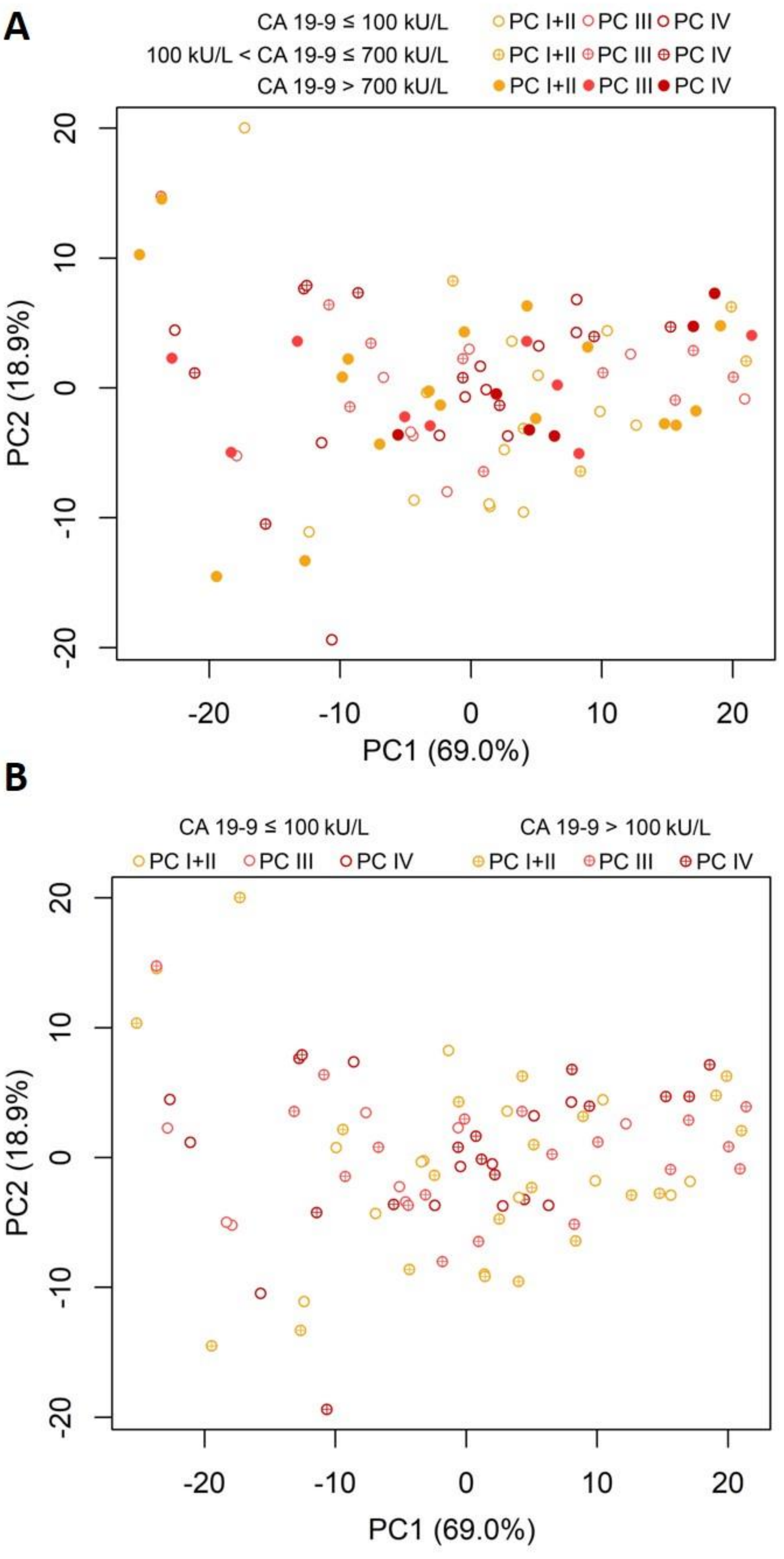

Figure S6. Principal component analysis of pancreatic cancer stages with graphical correlation with CA 19-9 marker, A) cut-off limit 100 kU/L and B) cut-off limits 100 and 700 kU/L.

Clinical examination of recent-onset diabetes mellitus patients

Table S7. Current health condition of all recent-onset diabetes mellitus patients included in the study.

Sample	Sample col.	DM diag.	Birth	Health condition
RODM 001	11/2016	11/2015	1960	post-acute pancreatitis, nonspecific parenchymal changes
RODM 002	11/2016	11/2015	1942	no clinical problems
RODM 003	12/2016	12/2015	1951	no clinical problems
RODM 004	12/2016	11/2016	1966	no clinical problems
RODM 005	12/2016	06/2014	1948	no clinical problems
RODM 007	01/2017	01/2016	1944	no clinical problems
RODM 008	01/2017	04/2016	1944	severe chronic pancreatitis
RODM 009	20/2017	20/2016	1952	no clinical problems
RODM 010	02/2017	02/2015	1950	no clinical problems
RODM 011	03/2017	03/2016	1956	04/2017 resection, early stage PC
RODM 012	04/2017	04/2016	1960	no clinical problems
RODM 013	04/2017	04/2015	1933	no clinical problems
RODM 014	04/2017	04/2015	1956	2017 resection duodenum / benign
RODM 015	04/2017	04/2016	1950	IPMN suspected
RODM 016	04/2017	04/2016	1949	no clinical problems
RODM 017	04/2017	04/2016	1956	no clinical problems
RODM 018	04/2017	04/2015	1961	no clinical problems
RODM 019	04/2017	04/2014	1957	no clinical problems
RODM 020	04/2017	10/2016	1951	no clinical problems
RODM 021	04/2017	04/2016	1952	no clinical problems
RODM 024	05/2017	05/2015	1956	no clinical problems
RODM 025	05/2017	05/2016	1941	no clinical problems
RODM 026	06/2017	06/2016	1951	no clinical problems
RODM 027	06/2017	06/2015	1942	no clinical problems
RODM 028	06/2017	06/2014	1952	no clinical problems
RODM 029	06/2017	06/2015	1953	no clinical problems
RODM 030	06/2017	06/2015	1933	no clinical problems
RODM 031	06/2017	06/2015	1965	no clinical problems
RODM 032	07/2017	05/2017	1946	2021 metastatic renal cancer, deceased 01/2022
RODM 033	07/2017	07/2016	1947	no clinical problems
RODM 034	07/2017	07/2014	1953	no clinical problems
RODM 035	07/2017	07/2016	1958	no clinical problems
RODM 036	07/2017	06/2017	1961	01/2018 resection, chronic pancreatitis with focal necrotic acinar tissue
RODM 037	08/2017	04/2017	1956	polymorbid patient with severe DM
RODM 038	11/2017	11/2015	1956	RODM 14, second sample
RODM 039	11/2017	11/2016	1959	no clinical problems, diag. bladder carcinoma
RODM 040	12/2017	12/2017	1943	no clinical problems, 2019 resection, IPMN
RODM 041	01/2018	01/2016	1951	no clinical problems
RODM 042	01/2018	01/2016	1951	no clinical problems

Sample	Sample col.	DM diag.	Birth	Health condition
RODM 043	01/2018	01/2017	1948	no clinical problems
RODM 044	02/2018	02/2017	1961	no clinical problems
RODM 045	02/2018	02/2016	1945	no clinical problems
RODM 046	02/2018	02/2017	1945	no clinical problems
RODM 047	03/2018	03/2018	1945	no clinical problems
RODM 048	04/2018	04/2018	1954	05/2018 resection, early stage PC
RODM 049	05/2018	05/2015	1936	no clinical problems
RODM 050	05/2018	03/2018	1948	no clinical problems
RODM 052	06/2018	05/2019	1959	no clinical problems
RODM 053	07/2018	05/2019	1957	no clinical problems
RODM 061	10/2018	10/2018	1954	ulcerative colitis, coeliac disease
RODM 065	11/2018	11/2017	1965	severe chronic pancreatitis
RODM 066	12/2018	12/2015	1957	infection in post pancreatic fluid collection
RODM 067	12/2018	12/2017	1934	no clinical problems
RODM 068	01/2019	01/2016	1968	2019 res. IPMN
RODM 069	02/2019	02/2018	1969	no clinical problems
RODM 070	02/2019	02/2016	1956	no clinical problems
RODM 071	03/2019	02/2019	1950	no clinical problems
RODM 072	02/2019	01/2020	1965	no clinical problems

Abbreviation: col., collection; diag., diagnosis; GIST, gastrointestinal stromal tumor; IPMN, intraductal papillary mucinous neoplasm; PC, pancreatic cancer; res., resection.
